# Type VI secretion system contributes to Enterohemorrhagic *Escherichia coli* virulence by secreting catalase against host reactive oxygen species (ROS)

**DOI:** 10.1371/journal.ppat.1006246

**Published:** 2017-03-13

**Authors:** Baoshan Wan, Qiufen Zhang, Jinjing Ni, Shuxian Li, Donghua Wen, Jun Li, Haihan Xiao, Ping He, Hong-yu Ou, Jing Tao, Qihui Teng, Jie Lu, Wenjuan Wu, Yu-Feng Yao

**Affiliations:** 1 Laboratory of Bacterial Pathogenesis, Department of Microbiology and Immunology, Institutes of Medical Sciences, Shanghai Jiao Tong University School of Medicine, Shanghai, China; 2 Department of Laboratory Medicine, Shanghai East Hospital, Tongji University School of Medicine, Shanghai, China; 3 State Key Laboratory for Microbial Metabolism and School of Life Sciences and Biotechnology, Shanghai Jiao Tong University, Shanghai, China; 4 College of Biological Engineering, Zhejiang University of Technology, Hangzhou, China; 5 Department of Infectious Diseases, Ruijin Hospital, Shanghai Jiao Tong University School of Medicine, Shanghai, China; University of São Paulo FMRP/USP, BRAZIL

## Abstract

Enterohemorrhagic *Escherichia coli* (EHEC) is one major type of contagious and foodborne pathogens. The type VI secretion system (T6SS) has been shown to be involved in the bacterial pathogenicity and bacteria-bacteria competition. Here, we show that EHEC could secrete a novel effector KatN, a Mn-containing catalase, in a T6SS-dependent manner. Expression of *katN* is promoted by RpoS and OxyR and repressed by H-NS, and *katN* contributes to bacterial growth under oxidative stress *in vitro*. KatN could be secreted into host cell cytosol after EHEC is phagocytized by macrophage, which leads to decreased level of intracellular reactive oxygen species (ROS) and facilitates the intramacrophage survival of EHEC. Finally, animal model results show that the deletion mutant of T6SS was attenuated in virulence compared with the wild type strain, while the deletion mutant of *katN* had comparable virulence to the wild type strain. Taken together, our findings suggest that EHEC could sense oxidative stress in phagosome and decrease the host cell ROS by secreting catalase KatN to facilitate its survival in the host cells.

## Author summary

The type VI secretion system (T6SS) is a specific macromolecular protein export apparatus, and widely distributed in Gram-negative bacteria. Generally, T6SS has been shown to play an important role in anti-bacterial competition and virulence to eukaryotic hosts. Enterohemorrhagic *Escherichia coli* (EHEC) can cause severe foodborne disease, including abdominal cramps and diarrhea that may progress to bloody diarrhea and hemolytic uremic syndrome. In the current study, we show that the T6SS of EHEC is involved in its intracellular survival and virulence in mice. Specifically, the novel effector KatN, a Mn-catalase identified in this work updates the general role of the T6SS in the pathogenesis of EHEC, in the context of oxidative stress in host cytoplasm. Combined with the biased distribution of *katN* in the T6SS-containing bacteria, our data suggest that KatN, as a new T6SS effector, is a key virulence factor in the pathogenesis of T6SS^+^ bacteria.

## Introduction

Enterohemorrhagic *Escherichia coli* (EHEC) is a globally important zoonotic pathogen capable of causing diarrhea, hemorrhagic colitis and hemolytic–uremic syndrome (HUS) [1]. A prominent feature of EHEC infection is its low infectious dose. A dose of 50–100 colony forming units (CFUs) of EHEC is sufficient to cause disease in healthy individuals [2]. EHEC consists of multiple serotypes, among which O157:H7 is the one most commonly linked to epidemic and sporadic diseases in humans throughout China, Japan, North America, and Europe.

For efficient colonization in human intestines, EHEC needs adhere to the follicle-associated epitheliums, which results in the rapid contact of EHEC with underlying macrophage cells [3,4]. Macrophages are important components of the host innate immune system, and the lamina propria of the large intestine is rich in macrophages. When the intestinal epithelial barrier is damaged and microorganisms cross the basement membrane, the macrophages may be the first line of host defense system. Macrophage cells produce and release reactive oxygen species (ROS) in response to phagocytosis or various stimuli [5]. ROS are short-lived molecules derived from incomplete reduction of oxygen metabolites that at high levels have a bactericidal function by damaging DNA, lipid, proteins, and membrane [6].

To survive, replicate, and disseminate throughout the body, bacterial pathogens, especially these intracellular bacteria must overcome the antimicrobial oxidative burst produced by macrophages. General ROS degradation enzymes including catalases, peroxidases, and superoxide dismutases are used by most bacteria to survive under oxidative stress [7]. *E*. *coli* possesses multiple distinct catalases to defend itself against oxidative stress, including hydroperoxidase I (HPI), KatG and hydroperoxidase II (HPII), KatE [8,9]. The alkyl hydroperoxide reductase complex, consisting of AhpC and AhpF, could catalyze the reduction of hydrogen peroxide in an NADH-dependent manner in *E*. *coli* [10]. Another plasmid-borne catalase-peroxidase, KatP, has been found to contribute to the complex gene network protecting EHEC from peroxide-mediated oxidative damage [11].

The intracellular survival of bacterial pathogens relies on the specialized secretory systems, which can inject bacterial effectors into the cytosol of host cells. The type VI secretion system (T6SS) is widely spread in both pathogenic [12] and non-pathogenic Gram-negative bacteria [13], and contributes to competition in bacterial communities by delivering bacteriolytic toxins to target cells [14]. Besides participating bacterial competition, T6SS is also involved in the pathogenicity of several Gram-negative pathogens [15–20].

More than 10 orthologs of known T6SS components are uncovered in genome-sequenced pathogenic *E*. *coli* strains by *in silico* analysis [21]. It has been shown that the T6SSs in both avian pathogenic *E*. *coli* (APEC) [22–24], enteroaggregative *E*. *coli* (EAEC) [16] contribute to bacterial virulence. In the previous study, we showed that T6SS is functional in neonatal meningitis-causing *E*. *coli* K1 (NMEC), and two Hcp family proteins participate in different steps of bacterial interaction with human brain microvascular endothelial cells (HBMEC) in a coordinate manner, e.g., binding to and invasion of HBMEC, the cytokine and chemokine release followed by cytoskeleton rearrangement, and apoptosis [19].

Our preliminary data showed that both EHEC strains EDL933 and Sakai contain the T6SS gene cluster. We thus question whether the T6SS gene cluster plays roles in EHEC infection of host cells and/or competition with other bacteria. In this study, we confirmed that the T6SS of EHEC strain EDL933 is functional, and a novel T6SS effector protein KatN was identified to be the catalase secreted by EHEC to antagonize ROS of eukaryotic host cells to evade host immune killing.

## Results

### Analysis of the T6SS gene cluster in EHEC genome

**Fig 1:**
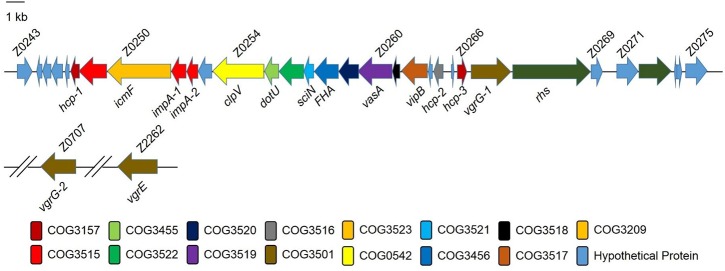
Schematic diagram of the genetic organization of the type VI secretion system (T6SS) in EHEC O157:H7 strain EDL933. Annotation of T6SS cluster genes of the EHEC strain EDL933. The database of Clusters of Orthologous Groups of proteins (COGs) was obtained from the National Center of Biotechnology Information (http://www.ncbi.nlm.nih.gov/COG/new/).

Genome sequence analysis showed that a 37-kb DNA fragment encoding a putative T6SS existed in the genome of EHEC strain EDL933. This fragment possessed several typical features of a composite pathogenicity island: it is associated with a tRNA-encoding gene *aspV* and harbors virulence genes. Annotation showed that this gene cluster contained z0250, z0254, z0255, *hcp-1*, *hcp-2*, *hcp-3*, and z0267, which are homologous to *vasK* (*icmF*-like), *vasH* (*clpV* homolog), *vasF* (*dotU* homolog), *hcp1*, *hcp2*, *hcp3*, and *vgrG* in *Vibrio cholerae*, respectively (Fig 1). These genes represented the core and conserved accessory components of the T6SS, suggesting the T6SS is functional in EHEC. The detailed annotation of EHEC T6SS ORFs was listed in the S1 Table. *In silico* analysis also showed that EHEC had another two *vgr* genes, *vgrG-2* (z0707) and *vgrE* (z2262) located in the other regions of genome. We then used our integrated database SecReT6 (http://db-mml.sjtu.edu.cn/SecReT6/) to compare the T6SS sequences [25], and found that the components and gene organization of the T6SS gene clusters were highly conserved in pathogenic *E*. *coli* strains compared with other bacteria including *V*. *cholerae* and *Salmonella* Typhimurium (S1 Fig).

### The T6SS of EHEC is functional and repressed by H-NS

We reanalyzed our previous RNA-seq data and found that the expression of T6SS *in vitro* was relatively low compared with other genes in EHEC genome [26]. The average reads per kilobase per million (RPKM) of T6SS gene cluster (z0243 to z0275) was 5.7, while the average RPKM of EHEC whole genome was 198.1 (S2 Fig). We then tested different culture conditions, including salt, pH, media and growth temperature, to stimulate the expression of T6SS *in vitro*. However, the qPCR results showed that these conditions could not induce the expression of T6SS (S3 Fig), suggesting T6SS may not be required for EHEC growth *in vitro*. Consistent with this speculation, the growth rate of the deletion mutant of T6SS was comparable to that of the parental strain (S4 Fig).

**Fig 2:**
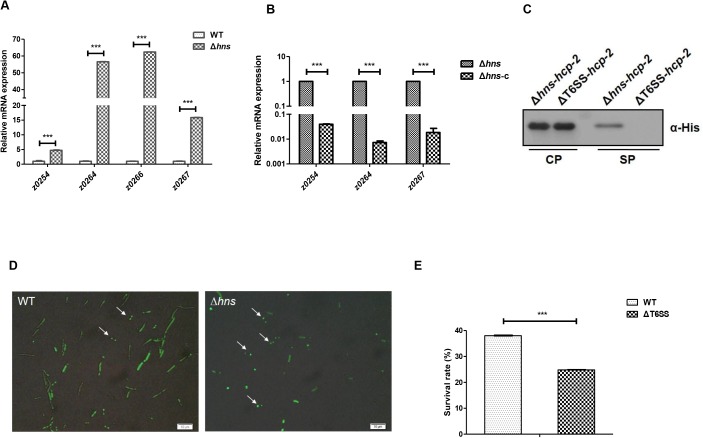
H-NS inhibits the expression of the T6SS genes of EHEC. (A) Transcription of T6SS genes in the wild type EHEC (WT) and *hns* deletion mutant (Δ*hns*). The WT and Δ*hns* were cultured to an OD_600_ = 1.0 in LB broth at 37°C. Cultures were harvested, and total RNA was isolated. The relative expression levels of z0254, z0264, z0267 were analyzed by qPCR. 16S rRNA was used as the reference gene. Error bars represented SD from at least three independent experiments. ***, P<0.001, ANOVA analysis. (B) The transcription of T6SS genes in the *hns* deletion mutant and complementation strains. Δ*hns* harboring either plasmid pACYC184 or pACYC184-*hns* were cultured to an OD_600_ = 1.0 in LB liquid at 37°C. Cultures were harvested followed by total RNA isolation. The relative expression levels of z0254, z0264(*hcp-2*) and z0267 were analyzed by qPCR. 16S rRNA was used as the reference gene. Error bars represented SD from at least three independent experiments. ***, P<0.001, ANOVA analysis. (C) The secretion of Hcp in EHEC. Δ*hns* and ΔT6SS harboring pQE80YX1-z0264 with a His-tag sequence fusion at the C-terminus were cultured to an OD_600_ = 1.0 in LB broth at 37°C. The pellet and supernatant fractions of the cultures were analyzed by Western blot using anti-His tag antibody. Three biological repeats were performed. (D) Formation of discrete ClpV–GFP foci. The WT and ΔT6SS harboring pQE80-z0254*-gfp* were cultured to an OD_600_ = 1.0 in LB liquid at 37°C. Cells were collected and resuspended in 1× PBS to an OD_600_ = 10. The resuspended cells were mixed with *E*. *coli* strain MG1655 at the ratio of 10:1 and transferred to the agarose pad. After incubation in 37°C for 30 min, ClpV-GFP foci were observed by fluorescence microscopy. 100 cells were analyzed, and the final percentages were obtained from three independent experiments. (E) The intracellular survival of the WT and ΔT6SS in RAW264.7 macrophages. RAW264.7 cells were incubated with the WT or ΔT6SS at an MOI of 10 for 30 min and then chased in the presence of 100 μg/ml gentamicin for 2 h to kill extracellular bacteria. Cells were then incubated for 20 h in the presence of 25 μg/ml gentamicin. Lysates were then plated to count viable intracellular bacteria. Percent bacterial survival was calculated based on viable counts (CFU/ml) relative to that at 2.5 h post-infection. Error bars represented SD from at least three independent experiments. ***, P<0.001, Student’s t-test analysis.

Because the highly conserved histone-like global repressor H-NS was found to repress the expression of T6SS in *V*. *cholerae* [27], *Edwardsiella tarda* [28] and *S*. Typhimurium [29], we speculate that H-NS may be a repressor of the T6SS in EHEC. We thus constructed the deletion mutant of *hns* in EHEC by replacing *hns* gene with chloramphenicol resistance cassette using λ Red recombination system [30]. Then, the bacterial total RNA was isolated, and the transcription level of T6SS genes was measured by quantitative real-time polymerase chain reaction (qPCR). The results showed that the expression of all the tested T6SS genes were upregulated in the deletion mutant of *hns* significantly (Fig 2A). Hcp-2 (Z0264) is a homologue of Hcp (Hemolysin co-regulated protein), which is an essential member of T6SS and involved in the assembly of the cogwheel-like protein complex in *V*. *cholerae* [31]. As a hallmark of T6SS, the transcription of *hcp-2* in the deletion mutant of *hns* increased by 60-fold compared with that of the parental strain. To exclude the potential polar effect of *hns* deletion, we used pACYC184 to deliver a copy of *hns* to the deletion mutant of *hns*. The qPCR result showed that the transcription of T6SS genes in the deletion mutant of *hns* was inhibited by *hns* complementation (Fig 2B).

The secretion of the T6SS hallmark effector protein Hcp is considered a reliable indicator of a functional T6SS [18,19]. We then used Western blot to investigate the secretion of Hcp to confirm the activity of T6SS and the repression of H-NS on the T6SS in EHEC. The deletion mutant of T6SS was constructed by replacing the 37 kb T6SS gene cluster with chloramphenicol resistance cassette. The mutant was verified by PCR and sequencing before further experiments. The Hcp-2 (Z0264) was fused by a His-tag sequence at C-terminus in the plasmid (pQE80-z0264), and the plasmid was transferred to the deletion mutants of T6SS or *hns* individually. After IPTG induction, the supernatants from these two strains were isolated and separated in SDS-PAGE. Western blot using anti-His antibody showed that Hcp-2 was expressed in the cytosol in both strains, but was only detected in the supernatant of the deletion mutant of *hns* (Fig 2C), suggesting that the T6SS was functional and could secrete hallmark effector protein Hcp-2 in EHEC.

Since the transcription of T6SS genes was upregulated in the deletion mutant of *hns*, we wonder whether the assembly of T6SS apparatus was also increased in this mutant. The assembly of an active T6SS can be visualized by fluorescent tagged ClpV, which is an AAA^+^ family protein that has been postulated to couple ATP hydrolysis to T6SS effector translocation. A functional T6SS apparatus results in a focused localization of ClpV, instead of diffused distribution [32]. We then fused GFP (green fluorescent protein) to the C-terminus of ClpV (Z0254) to visualize T6SS by co-incubating EHEC strains with *E*. *coli* K-12 strain MG1655 at a ratio of 10:1. The results showed that more *hns* mutant cells (47.0%) had GFP foci compared with the wild type strain (17.5%), indicating the deletion of *hns* could derepress the assembly of T6SS apparatus (Fig 2D). Interestingly, most GFP foci showed apparent polar localization in the deletion mutant of *hns*, which was contradictory to the central location of T6SS apparatus with bactericidal activity [33].

EHEC infection specifically results in the pathological attaching and effacing lesions of the follicle-associated epithelial layer [34], followed by rapid contact with underlying human macrophage cells. To test the role of T6SS in EHEC interaction with macrophages, we used RAW264.7 murine macrophage-like cells to study the intracellular survival of the wild type EHEC strain and the deletion mutant of T6SS. After 20 h incubation, the survival rate of the wild type strain in RAW264.7 cells was about 38.0%, while the survival rate of the deletion mutant of T6SS decreased dramatically to 24.8% (Fig 2E), suggesting that T6SS was involved in the intracellular survival of EHEC.

### Bactericidal activity of EHEC T6SS is not detected *in vitro*

**Fig 3:**
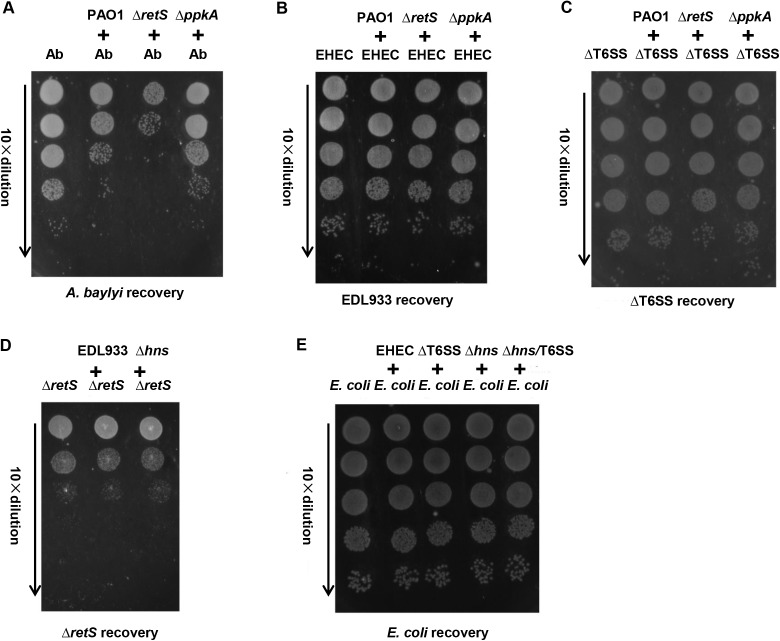
The T6SS of EHEC does not show antibacterial activity *in vitro*. (A) The positive control of competition assays. *A*. *baylyi* ADP1 was used as a prey strain to be killed by the predator strains (*P*. *aeruginosa* PAO1, Δ*retS* or Δ*ppkA*). The predator strains were individually mixed with *A*. *baylyi* ADP1 in 20:1 ratio, and 5 μl of the mixture was spotted on LB agar plate. After incubated at 37°C for 2.5 h, bacterial spots were cut out and the cells were resuspended in 1 ml 1× PBS. The suspensions were diluted serially in 1× PBS, and 5 μl of the suspensions was spotted on the selective LB agar plates, followed by 16 h incubation at 30°C. PAO1 indicates *P*. *aeruginosa* PAO1; Δ*retS* indicates *P*. *aeruginosa* PAO1 Δ*retS*, a T6SS-activated (T6SS^+^) strain; Δ*ppkA* indicates *P*. *aeruginosa* PAO1 Δ*ppkA*, a T6SS-inactivated (T6SS^-^) strain. EHEC T6SS^+^ (B) and T6SS^-^ (C) can not be killed by T6SS^+^ *P*. *aeruginosa*. (D) EHEC T6SS^+^ can not kill T6SS^+^ *P*. *aeruginosa*. Δ*hns* indicates EHEC Δ*hns*, a T6SS-activated strain. (E) T6SS^+^ EDL933 can not kill T6SS^-^ *E*. *coli* strain MG1655.

Recently, several findings reveal that T6SSs in some bacterial species have antagonistic bactericidal activity towards heterologous bacterial species [35–38]. The bactericidal activity of T6SS provides a strong competitive advantage against other bacteria in the host or the natural environments. As an intestinal pathogen, EHEC can colonize in the human intestine where a dense and diverse intestinal microbiota comprising a large amount of various bacteria exists [39]. It has been reported that *P*. *aeruginosa* could effectively kill *Acinetobacter baylyi* T6SS^+^ cells [33], so these two strains were included in the competition assay as the positive control. When mixed with *P*. *aeruginosa* Δ*retS*, a strain showing a dramatic increase in T6SS activity due to the derepression of T6SS by *retS* deletion, ~ 1000-fold fewer *A*. *baylyi* were recovered compared to that mixed with *P*. *aeruginosa* Δ*ppkA*, a T6SS defective strain (Fig 3A). However, *P*. *aeruginosa* Δ*retS* could not kill the wild type EHEC strain (Fig 3B) or the deletion mutant of T6SS, and *vice versa* (Fig 3C and 3D). It has been shown that certain T6SSs can kill T6SS negative (T6SS^-^) species [33]. We wonder whether EHEC could kill T6SS^-^ bacteria (e.g. *E*. *coli* K12 strain MG1655). As shown in Fig 3E, none of EHEC, Δ*hns*, ΔT6SS or the double deletion mutant (Δ*hns*/T6SS) could kill *E*. *coli* K12 strain, suggesting that EHEC did not have detectable bactericidal activity *in vitro*.

### Identification of T6SS effectors in EHEC

Since T6SS was critical to EHEC survival in macrophages (Fig 2E), we propose that T6SS may utilize unknown effector(s) to achieve its roles *in vivo*. The supernatants from the deletion mutant of T6SS and the WT cultures were isolated and purified by combining ultrafiltration membrane package and centrifugal filter [19], and separated in SDS-PAGE (S5A Fig). The quality of the supernatants was confirmed by Western blot using the antibody of RNA polymerase subunit alpha (RpoA), a cytosol marker of *E*. *coli* (S5B Fig). The supernatants were applied to liquid chromatography-tandem mass spectrometry (LC-MS/MS), and proteins that were only present in the supernatant of the wild type strain but not that of the T6SS deletion mutant were considered as potential T6SS effector candidates in EHEC. We repeated the above LC-MS/MS analysis using two sets of biological samples and found 46 and 31 T6SS effector candidates, respectively (S2 Table). Further analysis showed that three candidates, Z0873, Z1921 and Z5583 were detected by both LC-MS/MS performances, suggesting these three proteins were likely to be T6SS effectors in EHEC.

**Fig 4:**
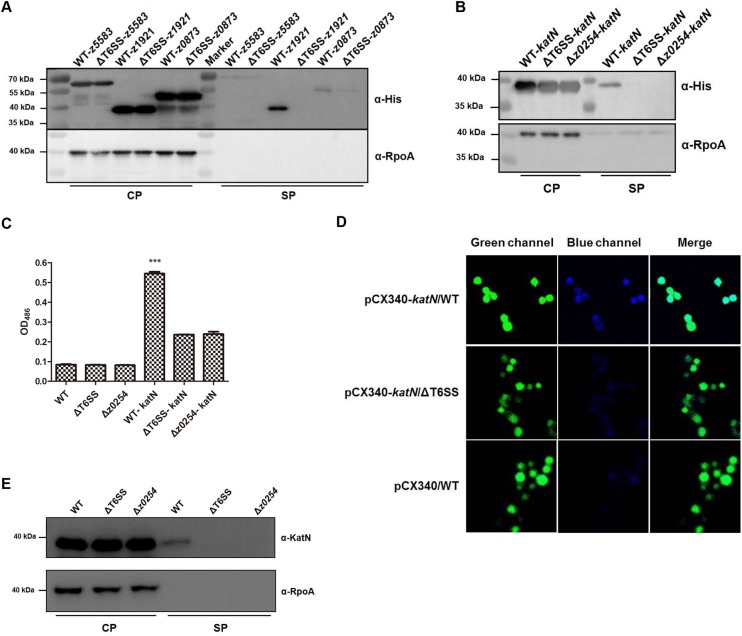
The secretion of KatN is dependent on T6SS in EHEC. (A) Verification of the T6SS effector candidates by Western blot. The WT and ΔT6SS bearing pQE80-z1921(*katN*) or z5583 or z0873 with a His-tag sequence fusion at the C-termini were cultured to an OD_600_ = 1.0 in LB broth at 37°C. The pellet and supernatant fractions of the cultures were analyzed by Western blot using the anti-His tag and anti-RpoA monoclonal antibodies. RpoA was used as an internal control. Three biological repeats were performed. (B) z0254 is required for the secretion of KatN. The WT, Δz0254 and ΔT6SS harboring pQE80-z1921(*katN*) with a His-tag sequence fusion at the C-terminus were cultured to an OD_600_ = 1.0 in LB broth at 37°C. The pellet and supernatant fractions of the cultures were analyzed by Western blot using the anti-His tag and anti-RpoA monoclonal antibodies. RpoA was used as an internal control. Three biological repeats were performed. (C) The β-lactamase fusion assay of KatN. The WT, Δz0254 and ΔT6SS bearing pCX340 or pCX-z1921(*katN*) were cultured to an OD_600_ = 1.0 in LB broth at 37°C. The β-lactamase activities in the supernatants of the cultures were detected by monitoring optical density change at 486 nm. The β-lactamase activity data (means ± SD) represented the results from duplicate samples of three biological repeats. ***, P<0.001, ANOVA analysis. (D) TEM-1 β-lactamase translocation assay. RAW264.7 cells were infected with EHEC strain EDL933 (WT) or ΔT6SS bearing pCX340 or pCX-*katN*, and visualized by fluorescence microscopy with excitation at 409 nm or 488 nm after being treated by CCF2-AM. Emission due to CCF2-AM can be viewed as green fluorescence at 520 nm, whereas disruption of CCF2-AM by Bla fusion protein activity results in emission at 447 nm (blue fluorescence). One representative field was shown for each strain, and the assay was conducted at least twice. (E) The secretion of KatN is confirmed by Western blot using the anti-KatN antibody. The WT, Δz0254, Δz0254 bearing pACYC184-z0254 were cultured to an OD_600_ = 1.0 in LB broth at 37°C. The pellet and supernatant fractions of the cultures were analyzed by Western blot using the anti-KatN and anti-RpoA antibodies. RpoA was used as an internal control. Three biological repeats were performed.

We next used His-tag fusion strategy to verify the secretion pathway of the three candidates. The plasmids harboring z0873, z1921 or z5583 with a His-tag sequence fusion at their C-termini were constructed and transformed to the T6SS deletion mutant or the wild type strain individually. The culture supernatants from these strains were isolated by trichloroacetic acid (TCA) precipitation [40] and separated in SDS-PAGE followed by Western blot using anti-His tag antibody. The results showed that all the three proteins were expressed in the bacterial cytosols, and only z0873 and z1921 could be secreted into supernatants. Because Z0873 was detected in the supernatants of both the WT and T6SS deletion mutant, we assumed that this protein was secreted in a T6SS-independent manner in EHEC and excluded for further analysis. Interestingly, Z1921 was only detected in the supernatant of the WT, but not in that of T6SS deletion mutant (Fig 4A), suggesting that the secretion of Z1921 was dependent on the T6SS in EHEC. Amino acids sequence analysis showed that Z1921 of EHEC shared 84% identity and 93% similarity with Mn-containing catalase KatN of *Salmonella enterica* [41], therefore Z1921 was named as KatN (S6 Fig).

We then transformed the plasmid harboring KatN-His tag fusion into the deletion mutant of z0254 (*clpV*). The supernatants of strains Δz0254, ΔT6SS and the WT bearing KatN-His tag fusion plasmid were isolated and applied to Western blot to check the secretion pathway of KatN. The result showed that the loss of z0254 alone or T6SS as a whole caused the failure of KatN secretion in EHEC (Fig 4B).

To consolidate these findings, we transformed pCX-*katN* (KatN-bla fusion was made by the insertion of the *bla* gene at the C-terminus of *katN*) into the WT and Δz0254 or ΔT6SS individually and examined the β-lactamase activity in the bacterial culture supernatants as described previously [19]. In this condition, the secretion of β-lactamase is dependent on the secretion of KatN. As expected, significant color change of nitrocefin was detected in the culture supernatant of WT-pCX*-katN*, while only marginal color alteration was observed in these of ΔT6SS-pCX-*katN* or Δz0254-pCX-*katN*, which may be due to the leaky expression of β-lactamase (Fig 4C). In addition, we also examined the translocation of the KatN fusions in infected RAW264.7 cells by using TEM-1 β-lactamase as a fluorescence-based reporter [42]. EHEC strains expressing the above-mentioned fusion proteins were used to infect RAW264.7 cells, and the KatN-bla fusion protein levels at the WT and ΔT6SS were comparable (S7 Fig). After 3 h incubation, the cells were washed and incubated for an additional 60 min with the β-lactamase substrate CCF2. The cells were then analyzed by a confocal microcopy with the simultaneous observation of the green fluorescence emitted by the CCF2-AM substrate and the blue fluorescence emitted by the cleaved CCF2-AM. Cells infected with ΔT6SS harboring pCX-*katN* fusions appeared dominantly green, indicating the absence of β-lactamase activity in these cells. In contrast, cells infected with EHEC expressing KatN fused to β-lactamase showed strong blue fluoresce signals (Fig 4D). The percentage of blues cells was about 76% in RAW264.7 cells infected by the WT EHEC bearing pCX-*katN*, while the percentages in control groups (WT-pCX340 and ΔT6SS-pCX-*katN*) were only about 33% (S8 Fig), indicating that KatN was efficiently translocated into the cells.

To avoid any effects that might result from overexpression of a plasmid-encoded gene, we then prepared polyclonal antibody of KatN and used this antibody to check the secretion of KatN in the supernatants of the deletion mutants of z0254, T6SS or the WT. The results again confirmed that KatN could only be detected in the supernatants of the WT, but not in these of the deletion mutants of z0254 or T6SS (Fig 4E). Furthermore, our data showed that the secretion deficiency of KatN due to the loss of z0254 could be restored by complementation (S9 Fig). Since T3SS of EHEC is responsible for the secretion of multiple effectors [43], we then tested the relationship between the secretion of KatN and T3SS, and results showed that the absence of a key T3SS gene, *escN*, did not disturb the secretion of KatN, indicating that T3SS was not involved in the secretion of KatN in EHEC (S10 Fig). Taken together, these data clearly demonstrated that KatN was secreted *via* T6SS in EHEC.

### Role and expression regulation of *katN*

**Fig 5:**
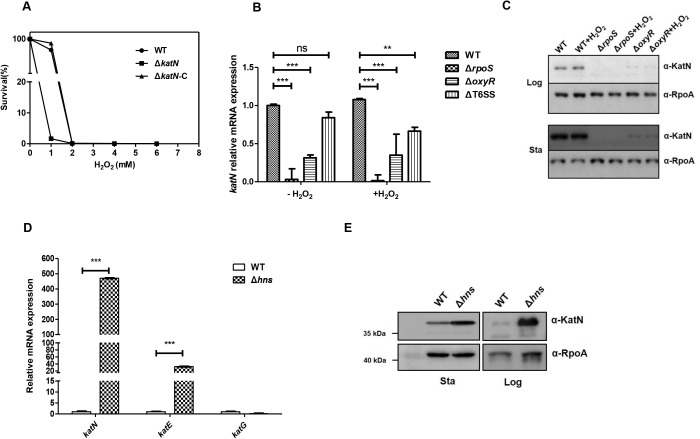
The role and transcriptional regulation of *katN*. (A) KatN contributes to the response of EHEC to oxidative stress. The WT, Δ*katN* and Δ*katN*-c were cultured in LB broth supplemented with H_2_O_2_ at final concentrations of 0, 1, 2, 4 and 6 mM at 37°C. The survival percentages were calculated by using the values OD_600_ of cultures after 7 h incubation. The measurements were repeated three times and a representative experiment was shown. (B) The transcription of *katN* is promoted by RpoS and OxyR. Logarithmic-phase (OD_600_ = 1.0) cultures (LB broth, 37°C) of the WT, Δ*rpoS*, Δ*oxyR* and ΔT6SS were divided into two aliquots followed by 1 mM H_2_O_2_ treatment or without H_2_O_2_ treatment. The cells were then harvested after 30 min and total RNA were isolated. The relative expression level of *katN* was analyzed by qPCR. 16S rRNA was used as the reference gene. Error bars represented SD from at least three independent experiments. **, P<0.01; ***, P<0.001; ns, Not significant, ANOVA analysis. (C) The expression of KatN is regulated by RpoS and OxyR. Logarithmic-phase (OD_600_ = 1.0) and stationary-phase (OD_600_ = 4.0) cultures (LB broth, 37°C) of the WT, Δ*rpoS* and Δ*oxyR* were divided into two aliquots followed by 1 mM H_2_O_2_ treatment or without H_2_O_2_ treatment. The cells were then harvested after 30 min and boiled with SDS sample buffer. The samples were separated in 12% SDS-PAGE followed by Western blot analysis using the anti-KatN and anti-RpoA monoclonal antibodies. RpoA was used as a loading control. Three biological repeats were performed. (D) KatN and other catalase genes expression in the WT and *hns* deletion mutant. Logarithmic-phase (OD_600_ = 1.0) cultures (LB broth, 37°C) of the WT and Δ*hns* were harvested for total RNA isolation. The relative expression levels of *katN*, *katE* and *katG* were analyzed by qPCR. 16S rRNA was used as the reference gene. Error bars represented SD from at least three independent experiments. **, P<0.01; ***, P<0.001, ANOVA analysis. (E) H-NS inhibits the expression of KatN. Logarithmic-phase (OD_600_ = 1.0) and stationary-phase (OD_600_ = 4.0) cultures (LB broth, 37°C) of the WT and Δ*hns* were harvested and separated on 10% SDS-PAGE. KatN expression was analyzed by Western blot using the anti-KatN and anti-RpoA monoclonal antibodies. RpoA was used as a loading control. Three biological repeats were performed.

Since KatN of EHEC may be a Mn-containing catalase, we then overexpressed and purified KatN to apparent homogeneity (S11A Fig) to check its catalase activity. The result showed that the specific activity of KatN is 268.3 U/mg protein (S11B Fig). Since KatN has a high catalase activity, we wonder whether it contributes to the EHEC response to oxidative stress. Thus, we used different concentrations of hydrogen peroxide to treat the WT, Δ*katN* and Δ*katN-*c, and monitored the bacterial growth. The absence of *katN* did not affect the bacterial growth in regular LB medium (S4 Fig), but caused the growth defect of EHEC under hydrogen peroxide concentrations from 1 mM to 2 mM (Fig 5A). This growth retardation could be restored by complementation of a copy of *katN*, indicating that *katN* contributed to EHEC response to oxidative stress *in vitro*.

It has been shown that OxyR is a principal regulator for hydrogen peroxide detoxification, and RpoS is a general stress response regulator at the stationary phase [44]. Since the expression of catalases KatE and KatG was regulated by OxyR and RpoS [45], we speculate that expression of *katN* might be regulated by these two regulators. The deletion mutants of *oxyR*, *rpoS* as well as the WT and ΔT6SS were treated by hydrogen peroxide, and qPCR was employed to determine the transcriptional levels of *katN* compared to mock treatment in these strains. The results of qPCR showed that both OxyR and RpoS were involved in the activation of *katN* regardless of the presence or absence of hydrogen peroxide. Specifically, RpoS was essential for *katN* transcription, and OxyR promoted the transcription of *katN* (Fig 5B). Morgan et al. demonstrates that KatG and AhpC are induced by hydrogen peroxide in *S*. Typhimurium [46], while our data showed that hydrogen peroxide did not induce the transcription of *katN* in EHEC, which is similar to the case of HPII KatE [47]. Moreover, the deletion of T6SS gene cluster did not affect the transcription of *katN*. The protein levels of KatN in the above conditions were determined by Western blot using anti-KatN antibody, showing that KatN protein levels were well correlated with the mRNA levels of *katN* in both log phase and stationary phase (Fig 5C).

As a global regulator, H-NS could inhibit the transcription of T6SS in EHEC. We then tested whether the transcription of *katN* and other catalase genes, *katG* and *katE* is also regulated by H-NS. The qPCR results showed that transcriptional level of *katN* increased by over 450-fold in the deletion mutant of *hns* compared with that of the WT (Fig 5D). Another catalase gene *katE* was upregulated in the deletion mutant of *hns*, however, transcription of *katG* was barely promoted in the deletion mutant of *hns*. The repression of H-NS on the expression of *katN* was further confirmed by Western blot using anti-KatN antibody (Fig 5E).

EHEC has multiple catalases to resist oxidative stress [11,48,49]. Given the previous evidence that KatN was secreted by T6SS, we further wonder whether EHEC could secrete other catalases through the same secretory apparatus for better intracellular survival. We then used His-tag strategy to determine the secretion pattern of all the catalases KatE, KatG, KatP and AhpC in EHEC. The results showed that all the catalases were expressed in the cytosols of the WT, ΔT6SS and Δz0254, but none of these catalases could be secreted (S12 Fig), suggesting KatN was the sole secreted catalase in EHEC up to date.

### KatN plays an important role for the survival of EHEC in macrophages

**Fig 6:**
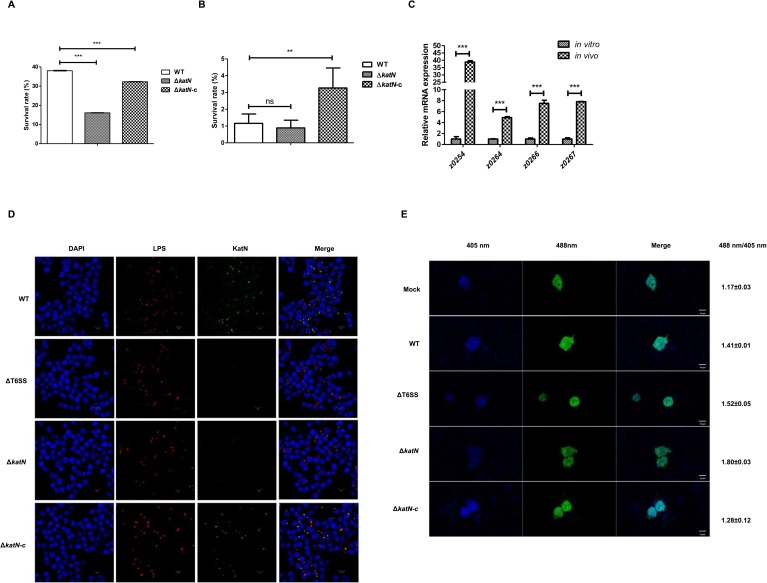
KatN is crucial for the survival of EHEC in macrophages. (A) The intracellular survival of the WT, Δ*katN* and Δ*katN-*c in RAW264.7 macrophages. RAW264.7 cells were incubated with the WT, Δ*katN* or Δ*katN* harboring pACYC184-*katN* (Δ*katN*-c) at an MOI of 10 for 30 min and then chased in the presence of 100 μg/ml gentamicin for 2 h to kill extracellular bacteria. Cells were then incubated for 20 h in the presence of 25 μg/ml gentamicin. Lysates were then plated to count viable intracellular bacteria. Percent bacterial survival was calculated based on viable counts (CFU/ml) relative to that at 2.5 h post-infection. All graphs displayed relative mean ± SD of at least three independent experiments. ***, P<0.001, ANOVA analysis. (B) The intracellular survival of the WT, Δ*katN* and Δ*katN-c* in primary peritoneal macrophages. Primary peritoneal macrophage cells isolated from BALB/c mice were incubated with the WT, Δ*katN* or Δ*katN*-c, then treated as described above. Error bars represented SD from at least three independent experiments. **, P<0.01; ns, Not significant, ANOVA analysis. (C) The expression of the T6SS genes and *katN* in macrophages. RAW264.7 cells were infected with the WT at an MOI of 50. After 8 h incubation, cells were lysed and total bacterial RNA was isolated. The relative expression levels of z0254, z0264, z0266, and z0267 were analyzed by qPCR. 16S rRNA was used as the reference gene. Error bars represented SD from at least three independent experiments. ***, P<0.001, ANOVA analysis. (D) KatN is secreted into the cytoplasm of macrophages *via* T6SS. RAW264.7 cells were infected with the WT or its derived mutants (ΔT6SS, Δ*katN*, Δ*katN*-c) at an MOI of 10. After 40 min incubation, cells were fixed with 4% para-formaldehyde and incubated with anti-O157 LPS and anti-KatN antibodies. Cell nuclei were counterstained with DAPI. Samples were analyzed by a laser-scanning confocal microscope. The red spots represented bacterial signals and the green spots represented KatN signals in the cytoplasm of RAW264.7 cells. Three biological repeats were performed. Scale bar equals 10 μm. (E) KatN contributes to the decrease of ROS level in macrophage cells. Hyper3-transfected RAW264.7 cells were infected with the WT or its derived mutants (Δ*katN* and Δ*katN-*c) at an MOI of 10. After 40 min incubation, cells were quantified at excitation wavelengths of 405 nm and 488 nm by a laser-scanning confocal microscope. The ratios of 488 nm/405 nm were the averages of at least 10 cells. At least three biological repeats were performed. SD was derived from at least three independent experiments. **, P<0.01, ANOVA analysis. Scale bar equals 10 μm.

EHEC infection specifically results in the pathological attaching and effacing lesions of the follicle-associated epithelium [34], followed by its rapid contact with underlying human macrophage cells. To test the role of *katN* in EHEC interaction with macrophages, we used murine macrophage cell line RAW264.7 to study the survival of the wild type EHEC strain and its derived mutants. After 20 h incubation, the survival rate of the wild type strain in RAW264.7 cells is about 38.0%, while the survival rate of the deletion mutant of *katN* decreased dramatically to 16.1%. The survival defect of the deletion mutant of *katN* could be restored to by introducing a copy of *katN* (Fig 6A). This observation was confirmed by using primary peritoneal macrophage cells (Fig 6B), suggesting that *katN* contributed to the survival of EHEC in macrophage cells.

Since T6SS played an important role in the survival of EHEC in macrophage cells (Fig 2E), while the expression of T6SS was relatively low *in vitro* (S2 Fig), we speculate that the expression of T6SS may be induced when EHEC was phagocytized. To test this hypothesis, we infected RAW264.7 cells with the wild type EHEC strain and isolated the intracellular bacteria, followed by total RNA purification and qPCR analysis. The result showed that the expression of T6SS was upregulated dramatically. The transcriptional levels of all the tested genes including z0254, z0264, z0266 and z0267 were increased by 40, 8, 7 and 8 fold compared with these of the genes *in vitro*, respectively (Fig 6C). We also studied the transcriptional levels of all the catalases genes in EHEC including *katN*, *katE*, *katG*, *katP*, and *ahpC* and found that all catalases genes were upregulated by 4 fold to 67 fold in the intracellular bacteria except *ahpC* (S13 Fig). These data suggested that after phagocytosis by macrophage, EHEC induced the expression of T6SS and catalases for a better intramacrophage survival.

Next, we want to give direct evidence that KatN is secreted when EHEC is phagocytized by macrophage. To address this question, we used the wild type EHEC strain, the deletion mutant of T6SS, the deletion mutant of *katN*, and *katN* complementation strain by introducing KatN-expression plasmid in the *katN* deletion background (Δ*katN*-c) to infect RAW264.7 cells. Cells were then stained by DAPI (4’,6’-diamidino-2-phenylindole), anti-EHEC LPS antibody or anti-KatN antibody, followed by corresponding fluorescent-dye conjugated secondary antibodies detection. The anti-EHEC LPS antibody indicated that the infection rates of RAW264.7 cells by different EHEC strains were similar. As we expected, the WT EHEC-infected RAW264.7 cells showed strong KatN fluorescence signal in the cytosol, while neither ΔT6SS- nor Δ*katN-*infected RAW264.7 cells showed KatN signal. When a copy of *katN* was introduced into Δ*katN*, the signal of KatN reappeared in the cytosol of RAW264.7 cells (Fig 6D).

As innate immune cell species, macrophages can generate ROS to kill some types of engulfed bacteria [5]. Since EHEC could secrete catalase KatN into host cell cytosol *via* T6SS, we speculate that EHEC may utilize the secreted KatN to antagonize host cell ROS to escape from killing by phagocytic cells. Therefore, we used HyPer-3, a genetically encoded fluorescent indicator to determine intracellular ROS level in macrophages. Specifically, ROS can change the excitation spectrum of HyPer-3 with an excitation maximum at 405 nm for the reduced and 488 nm for the oxidized state, and the ratio of signals at 488 nm and 405 nm presents the intracellular ROS level [50]. In other words, higher ratio of signals at 488/405nm indicates a more oxidized intracellular environment, or high ROS stress. After 1 h incubation, the ratio between both channels (488 nm/405 nm) in uninfected cells was 1.17±0.03, while the ratio in the wild type EHEC-infected cells increased to 1.41±0.01. As we expected, the ratio of 488 nm/405 nm of RAW264.7 cells infected by the deletion mutant of T6SS increased to 1.52±0.05 (*vs* that of WT-infected cells: P<0.01) (Fig 6E). Interestingly, the ratio of 488 nm/405 nm in the deletion mutant of *katN*-infected cells reached 1.80±0.03 (*vs* that of WT-infected cells: P<0.001), while introduction of *katN* in the deletion mutant of *katN* (Δ*katN*-c) decreased the ratio of 488 nm/405 nm to 1.28±0.12 (*vs* that of Δ*katN*-infected cells: P<0.001). To confirm this observation, we used DCFH-DA (2,7-dichlorodi hydrofluorescein diacetate) to directly monitor the production of intracellular ROS [51]. We infected RAW264.7 cells with the deletion mutant of *hns* (Δ*hns*), double deletion mutant of *hns* and *katN* (Δ*hns*/Δ*katN*), and *katN* complementation strain (Δ*hns*/Δ*katN*-c). After incubation and washing, DCFH-DA was applied to the cells followed by flow cytometry analysis. As we expected, the absence of *katN* increased the ROS level of RAW264.7 cells, which could be reverted by introducing a copy of *katN* (S14 Fig). These data showed that EHEC could utilize T6SS to secrete catalase KatN into host cells to decrease the ROS level and facilitate bacterial intracellular survival.

### T6SS contributes to EHEC virulence in mouse model

**Fig 7:**
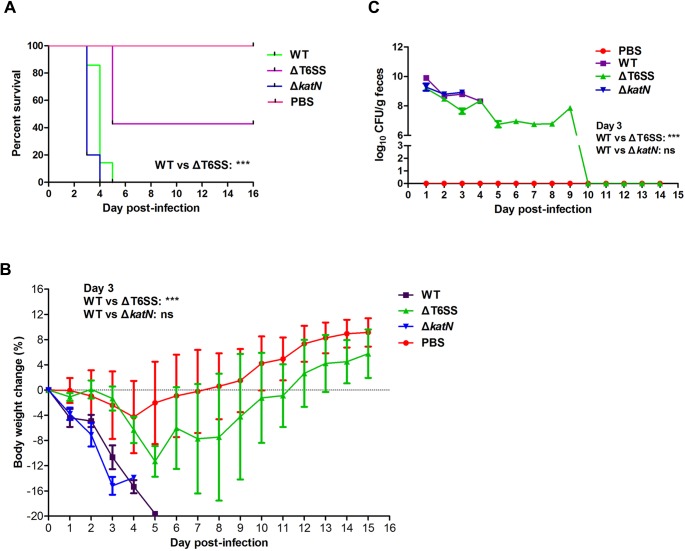
T6SS contributes to EHEC virulence in mouse model. (A) ΔT6SS has lower virulence in mice model compared with the WT. The streptomycin pre-treated BALB/c mice were intragastrically infected with the WT or its derived mutants at the inoculation of 10^10^ CFU. The percentage of surviving animals (8 mice per group) on each day was calculated. ***, P<0.001, Log-rank analysis. (B) Body weight changes of BALB/c mice infected by the WT, ΔT6SS or *ΔkatN*. The average body weight change percentage of surviving animals (8 mice per group) on each day was calculated. Error bars represented SD from at least three independent experiments. ***, P<0.001; ns, Not significant, ANOVA analysis. (C) The colonization of the WT, ΔT6SS or *ΔkatN* in mice gut. At the indicated times, fecal samples were collected, homogenized, diluted and plated on SMAC agar plates to determine the numbers of the WT or its derived mutants. The log_10_ means of CFU per gram of feces for each group of 8 mice are presented for each time point. Error bars represented SD from at least three independent experiments. ***, P<0.001; ns, Not significant, ANOVA analysis.

To determine the contribution of T6SS to EHEC virulence in mouse model, the WT EHEC, ΔT6SS or Δ*katN* strains were used to infect mice, respectively. Groups of 8 BALB/c mice were infected by oral gavage with 7×10^10^ CFU bacteria or Phosphate-buffered saline (PBS), and monitored for survival over a 16-day period. Mice infected with the WT or Δ*katN* appeared ruffled fur immediately after administration and began dying both at day 3 post-infection, and were all dead within 5 days. However, 40% of ΔT6SS-infected mice were still alive (vs WT: P<0.001, Log-rank analysis) and showed no infection-associated morbidity such as ruffling of fur and wasting (Fig 7A). We also monitored the mice body weight during the whole experiment period, and the result showed that the WT- and Δ*katN-*infected mice lost body weight dramatically after administration (vs ΔT6SS: P<0.001, at day 3, Student’s t test). Instead, ΔT6SS-infected mice lost body weight slightly compared with PBS control, and started to gain body weight from day 5 post-infection as PBS control (Fig 7B).

The bacterial load in feces was determined from day 1 to day 15 post-infection. The result showed that the colonization of all the three EHEC strains in the feces kept decreasing from day 1 post-infection. The bacterial burden in feces of ΔT6SS-infected mice was 7.6×10^7^ CFU, while the bacterial burden in feces of the WT- or Δ*katN*-infected mice were 8.8×10^8^ CFU and 9.0×10^8^ CFU (WT or Δ*katN* vs ΔT6SS: P<0.001 at day 3), respectively on day 3 post-infection (Fig 7C). After 10 days post-infection, EHEC strains could not be detected in the feces of ΔT6SS infected mice, indicating that the bacteria were completely cleared from the gut.

## Discussion

T6SS has been shown to be widely distributed in up to 25% of Gram-negative bacteria [21]. Although most of these bacteria possess only one single T6SS gene cluster, some species harbor multiple distinct T6SS copies. For example, five phylogenetically distinct T6SS loci have been identified in *S*. *enterica* [52], *Burkholderia pseudomallei* and *Burkholderia thailandensis* display six and five T6SSs, respectively [53], and *P*. *aeruginosa* possesses three T6SS clusters [54]. The distribution and copy number of T6SS in *E*. *coli* are various. APEC and EAEC contain up to 3 phylogenetically distinct T6SS clusters [55], while UPEC strain CFT037 only harbors one set of T6SS locus. There is one T6SS gene cluster in EHEC strains of Sakai and EDL933. Although the number of T6SS gene cluster is identical in UPEC and EHEC, the sequence and organization of their T6SS genes are distinct, suggesting the origin of T6SS is highly complicated.

T6SS has been extensively explored in bacterial antagonism in various environments. T6SS-dependent bactericidal activity was fulfilled by injecting an array of toxins to hydrolyze the prey cell wall. In this study, we found that both the wild type EHEC strain and the deletion mutant of *hns* (a T6SS activating strain) failed to kill T6SS^+^ or T6SS^-^ bacteria (Fig 3D and 3E), suggesting that T6SS in EHEC does not have antibacterial activity *in vitro*. At this stage, we can not rule out the possibility that EHEC may have bactericidal activity *in vivo*. EHEC is a zoonotic pathogen that thrives in the rumen of cattle [56], we then used bile salt supplement to mimic the human gastrointestinal tract environment, and found that the transcription levels of T6SS genes were dramatically induced by bile salt supplement (S15A Fig). Moreover, we found that T6SS contributed to the resistance of EHEC to bile salt (S15B Fig). Considering the expression level difference of T6SS between *in vitro* and *in vivo* (Fig 6C), one might postulate that with an enhanced T6SS expression, EHEC may replicate and target host microbiota in a T6SS-mediated manner for better survival and competition with other bacteria and protozoans in the cattle rumen.

Since T6SS was initially identified as a virulence factor in *E*. *tarda* in 2004 [57], it has been associated with bacterial virulence-related phenotypes, including adhesion, invasion, cytoskeletal alteration, intracellular survival, cytotoxicity, and host response, suggesting T6SS is indeed crucial for pathogen virulence [15–20]. As a protein secretion machine, T6SS in pathogenic bacteria is supposed to secrete an array of effectors to mediate bacterial interaction with eukaryotic cells. The involvement of EHEC T6SS in virulence was proved in this study (Figs 2E and 7A). Although Shiga toxin is considered to be the major virulence factor of EHEC [56], we found that deletion of T6SS did not disturb the expression and secretion of Shiga toxin (S16 Fig), indicating that T6SS contributed to the pathogenesis of EHEC independent of Shiga toxin activity.

The T6SS-dependent effectors remain elusive. There are few virulence-related effectors identified in the past decade except canonical Hcp and Vgr family effectors, which were discovered more than ten years ago [18]. Up to now, only four non-canonical T6SS effectors were reported, including EvpP from *E*. *tarda* [58], VasX from *V*. *cholerae* [59,60], PldB from *P*. *aeruginosa* [61], and TecA in *B*. *cenocepacia* [62]. In this study, we identified a catalase KatN as a novel T6SS effector in EHEC, and this effector could decrease the ROS level of host cell to facilitate EHEC survival in phagocytic cells. EHEC possesses multiple catalases and peroxidases for defense against oxidative stress, including KatE, KatG, KatP, and alkyl hydroperoxide reductase AhpC [8,9,11]. AhpC and KatG are regulated by both OxyR and RpoS [46,63,64]. KatE is only induced by RpoS during the switch from exponential to stationary growth [47]. We found that KatN is also regulated by both OxyR and RpoS (Fig 5B), suggesting a complicated regulatory network in response to oxidative stress in EHEC.

Although EHEC has several catalases, it seems these catalases are not redundant and play protective roles under different circumstances. Both KatG and KatE scavenge hydrogen peroxide in *E*. *coli*, and KatG is the major protective enzyme when AhpC is saturated by high level of hydrogen peroxide [65]. Our previous RNA-Seq data showed that RPKMs of *ahpC* and *katG* are 3471.8 and 618.9, respectively, which are much higher than the average RPKM of whole genome [26]. Considering the RPKM of *katN* is 4.0 under *in vitro* condition, and the transcriptional level of *katN* in macrophage cells was increased by more than 10 fold (S13 Fig), we postulate *katN* is not critical for EHEC response to oxidative stress *in vitro* but rather plays an important role in the interaction between EHEC and the host phagocytic cells. The survival defect of *katN* deletion strain in macrophage clearly supported this hypothesis. The similar phenotype of Δ*katN* as wild type strain in mouse model suggests other catalase(s) may play redundant roles *in vivo*.

We analyzed the distribution of *katN* in sequenced bacterial genomes and found that most pathogenic *E*. *coli* strains contain *katN*, and non-pathogenic *E*. *coli* strains do not possess *katN*. Moreover, *katN* and z1922, z1923 and z1924 consist of an operon in the genome of EHEC strain EDL933, which is highly homologous to the *yciGFEkatN* operon in *S*. Typhimurium [41]. When we extended the searching range from *E*. *coli* to other species, the results showed that most *katN* genes were linked to the operon *yciGFE* and located on a cryptic prophage (CP-933X), suggesting *katN* was acquired *via* horizontal gene transfer in EHEC. Further analysis showed that about 130 sequenced bacterial genomes contain *katN*, while most of them (100 of 130) were distributed in the T6SS containing bacterial species (S17 Fig), suggesting a co-existence of T6SS and *katN* in the pathogenic bacterial species. We further analyzed the distribution of *katE*, *katG*, *ahpC* and *katP*, and found that *katE*, *katG* and *ahpC* were highly conserved and wildly distributed in the bacterial genomes, while *katP* was only present in 47 species, and the distributions of the above genes were not correlated with these of T6SS clusters.

It has been reported that some bacteria species in human gut can induce rapid, physiological generation of ROS to regulate host immune function, intracellular signalling, and cytoskeletal dynamics [66–70]. For example, commensal bacteria, *Lactobacillus* can stimulate NADPH oxidase I to promote ROS generation in the gut [71]. On the other hand, intestinal pathogens must overcome the intestinal ROS barrier to colonize in the gastrointestinal tract to cause infection and disease. For instance, OxyR and two catalases are critical for scavenging environmental ROS to facilitate *V*. *cholerae* growth and zebrafish intestinal colonization [72]. The present work showed that EHEC could secrete catalase KatN in both cell contact-independent and contact-dependent manners. Therefore, we speculate that once entered into the intestine, EHEC might use T6SS to secrete catalase KatN to hydrolyze ROS around bacterial cell to form a low ROS level niche and facilitate EHEC population growth, further infection and development of disease.

## Materials and methods

### Strains, cell lines and growth conditions

The bacterial strains and plasmids used in this work were listed in S3 Table. *E*. *coli* O157:H7 strain EDL933 was used as the wild type EHEC strain. All mutants derived from strain EDL933 were constructed by λ Red recombinase system. *E*. *coli* BL21 (DE3) was used to overexpress KatN. *E*. *coli* DH5α was used for the cloning procedures. Mouse macrophage-like RAW264.7 cells obtained from Cell Resource Center of Shanghai Academy of Sciences, Chinese Academy of Sciences were used in this study. Bacteria were cultured routinely at 37°C in Luria Bertani (LB) medium unless noted otherwise. Cell lines were cultured in DMEM (10% FBS) at 37°C under 5% of CO_2_. Antibiotics were used at the following concentrations: ampicillin 50 μg/ml; gentamicin 100 μg/ml; chloramphenicol, 30 μg/ml; kanamycin, 50 μg/ml; streptomycin 200 mg/ml; tetracycline, 10 μg/ml.

### Identification of T6SS by *in sillico* analysis

The core of T6SS is composed of 13 proteins. Genes encoding these proteins were chosen as baits in sequential BLASTN, BLASTX and BLASTP to identify homologues in the genome of EHEC strain EDL933 (e-value less than 10^−5^).

### Plasmids and strains construction

The genomic DNA of *E*. *coli* was extracted using Easy-DNA kit (Invitrogen, Carlsbad, CA). The genes mentioned in this manuscript were amplified with the corresponding primers listed in S4 Table using the genomic DNA of EHEC strain EDL933 as template. The PCR products were digested with the corresponding restriction enzymes and then cloned into the appropriate vectors predigested by same restriction enzymes. The deletion mutant strains were constructed by the method described by Datsenko and Wanner [30]. Briefly, the EHEC strain EDL933 was transformed with the pKD46, which contains genes coding for the arabinose-induced λ Red recombinase system that promotes recombination between linear pieces of DNA (PCR products) and the host chromosome. The recombination is based on short stretches of homology (50 nucleotides) on the linear DNA to the site of recombination. The PCR products for knocking out the target genes were amplified, gel extracted, and electroporated into competent strain EDL933 containing pKD46 prepared with the presence of arabinose. The deletion mutants were screened by the antibiotics and were verified by PCR using primers adjacent to the gene region and followed by sequencing.

### Identification of T6SS substrates

The secreted proteins were isolated by a previously method with some modifications [19]. The overnight inoculums of strain EDL933 and its derived mutants were diluted 1:100 into 1 L M9 minimal medium supplemented with 44 mM NaHCO_3_, 8 mM MgSO4, 0.4% glucose, and 0.1% Casamino Acids for further growth to an OD_600_ of 0.8, at 37°C. The supernatant was collected by centrifugation twice at 20,000 g for 15 min and then filtered through a 0.2 μm filter. The filtered supernatant was condensed 100 fold *via* Vivaflow 50 system, according to the manufacturer’s instruments (Sartorius). The condensed supernatant was centrifuged at 25,000 g for 15 min to remove insoluble salts. The cleaned supernatant was further condensed by Amicon Ultra system at a cutoff of 3 kDa (Millipore). The resulting sample with a volume of about 100 μl were mixed with 30 μl of 5× SDS-PAGE Sample buffer and boiled for 5 min. The samples were applied to SDS-PAGE, Western blot or LC-MS/MS analysis.

### Western blot

Western blot was carried out according to standard procedures. Briefly, proteins were resolved on 12% SDS-PAGE, transferred to PVDF membranes. 50 mM Tris-HCl (pH 7.5) with 150 mM NaCl, 0.5% (V/V) Tween-20 (TBST) and 5% skim milk was used for blocking the PVDF membranes and diluting the antibodies. After blocking, membranes were probed with the anti-KatN (rabbit-derived) antibody or anti-RpoA (mouse-derived) antibody or anti-His tag (mouse-derived) antibody over night at 4°C. Membranes were washed with 1× TBST 3 times for 10 min each, and probed with appropriate secondary antibody.

### β-Lactamase assay

The β-Lactamase assay was performed as previously described [19], with some modifications. Briefly, EHEC strain EDL933 and its derived mutants bearing pCX340 or pCX-*katN* were grown overnight in LB medium with 10 μg/ml tetracycline and 0.25 mM IPTG at 37°C. The cultures were centrifuged at 12,000 g for 15 min, and the supernatants were collected. Five microliters of nitrocefin (Calbiochem) stock solution (1 mM) was added to 95 μl supernatant of each sample, then the mixture was incubated at room temperature for up to 15 min to allow red color to develop. Spectrophotometric assays for β-lactamase were carried out by measuring changes in absorbance at 486 nm.

### KatN purification

For purification of KatN, pQE80YX1-*katN* was constructed and verified by sequencing. The plasmid was introduced into *E*. *coli* strain BL21 (DE3), and the cells were grown in LB medium containing 100 μg/ml ampicillin at 37°C with shaking. IPTG was added to a final concentration of 0.1 mM at the time point when the absorbance of the culture at OD_600_ reached 0.6–0.8. The culture was continuously incubated for another 3–4 h. The cells were harvested by centrifugation and disrupted by ultrasonication (Sonics, USA). The supernatant was collected by centrifugation at 20,000 x g for 40 min at 4°C, and supplemented with imidazole at a final concentration of 10 mM. KatN was purified by the ÄKTA system (GE Health, USA). His-tag resin beans were washed with 10× volume binding buffer A (20 mM sodium phosphate, 500 mM NaCl and 20 mM imidazole, pH 7.5), recombinant protein was eluted using 10× volume of buffer B (20 mM sodium phosphate, 500 mM NaCl and 500 mM imidazole, pH 7.5). The protein was checked by SDS-PAGE before shock-frozen storage at -80°C. KatN concentration was determined by the Bradford protein assay (Bio-Rad, USA). Bovine serum albumin (BSA) was used as the protein concentration standard.

### KatN antibody preparation

The KatN protein tagged with hexa-histidine (6× His) was overexpressed and purified by nickel affinity chromatography. The purified KatN was used to immunize rabbits for the production of antibodies.

### Measurement of KatN catalase activity

Catalase activity was measured by using the Catalase Assay Kit (Beyotime) according to the manufacturer’s instruction. The bovine liver catalase (Sigma Aldrich, USA) was used as a positive control.

### Resistance to H_2_O_2_

The overnight cultures of strain EDL933 and its derived mutants were diluted and transferred into fresh LB medium to reach OD_600_ value of 1.0. To test the resistance to H_2_O_2_, bacteria cultures were diluted 100 times in 5 ml LB broth supplemented with 0 mM, 1 mM, 2 mM, 4 mM, 6 mM or 100 mM H_2_O_2_. After incubation at 37°C, 220 rpm for 7 h, the resistance to H_2_O_2_ was measured by determining the values of OD_600_.

### Quantitative real-time PCR (qPCR) assay

Briefly, the total RNA was isolated by RiboPure-Bacteria kit (Ambion, USA), and the concentration was determined by measuring the A260. Three microgram of total RNA was reverse-transcribed into cDNA by using Super Script III First-Strand Synthesis System for RT-PCR (Invitrogen, USA). Primers for qPCR were listed in S4 Table. Samples were run in triplicates and amplified using SYBR Premix Ex Taq II (TAKARA) in the 7500 fast Real-Time PCR System. The relative transcriptional level was determined by the methods of 2^−ΔΔCt^ [73]. 16S rRNA was used as a reference gene.

### Bacterial competition assay

Bacterial competition assays were performed according to the method previously described, with minor modifications [33]. The gentamicin-resistant plasmid was transformed into strain EDL933 or *P*. *aeruginosa* PAO1 for bacterial competition assays; *A*. *baylyi* ADP1 was spontaneous streptomycin resistant mutant, *E*. *coli* K12 MG1655 was kanamycin resistant derivative mutant. Overnight cultures of bacteria (EHEC EDL933, *P*. *aeruginosa* PAO1 or *A*. *baylyi* ADP1) were washed by LB and diluted 100 times into fresh LB broth and cultivated to OD_600_ ~ 0.8–1.0. Cells were washed by 1× PBS and enriched to OD_600_ ~ 10. Then, cells were mixed in 20:1 ratio, and 5 μl of the mixture was spotted on LB agar plate. After incubated at 37°C for 2.5 h, bacterial spots were cut out and the cells were resuspended in 1 ml 1× PBS. The suspensions were diluted serially in 1× PBS, and 5 μl of the suspensions was spotted on selective LB agar plates (gentamicin for strain EDL933 and *P*. *aeruginosa* PAO1, streptomycin for *A*. *baylyi* ADP1), followed by 16 h incubation at 30°C. Antibiotic concentrations were gentamicin, 15 μg/ml; streptomycin, 100 μg/ml; kanamycin, 10 μg/ml. At least three biological replicates were performed.

### Bacterial survival in macrophage cells

The RAW264.7 cells were maintained in DMEM supplemented with 10% fetal bovine serum (FBS) for survival experiment [73,74]. The RAW264.7 cells were seeded at 2×10^5^ cells per well in a 24-well tissue culture plate and cultured at 37°C under 5% of CO_2_ overnight. Then, the RAW264.7 cells were infected with 5×10^6^ cells of EHEC strain EDL933 and its derived mutants (MOI = 10). The plate was centrifuged briefly (400 g, 5 min) to synchronize the infection and incubated for 30 min (0 h) at 37°C under 5% of CO_2_. The cells were washed three times with PBS and fresh DMEM-10% FBS containing 100 μg/ml gentamicin was added to kill extracellular bacteria. After incubation for 2 h at 37°C under 5% of CO_2_, the cells were washed with PBS three times and lysed in 0.025% SDS, and then diluted with PBS for CFU counting on LB agar plates. To assess intracellular growth, the DMEM-10% FBS containing 100 μg/ml gentamicin was replaced with DMEM-10% FBS containing 15 μg/ml gentamicin and parallel cell cultures were analyzed for viable bacteria after 24 h incubation at 37°C under 5% of CO_2_.

### Measurement of intracellular gene expression

The transcription of T6SS genes of EHEC in RAW264.7 cells was analyzed by qPCR. In brief, 2×10^5^ RAW 264.7 cells were infected with 1×10^7^ EHEC strain EDL933 (MOI = 50) at 37°C under 5% of CO_2_. After incubation for 1 h, excess bacteria were washed off with PBS, and the infected cells were incubated with DMEM (10% FBS) at 37°C under 5% of CO_2_ for 8 h. The EHEC strain EDL933 was incubated individually with DMEM (10% FBS) at 37°C under 5% of CO_2_ for 8 h as an uninfected control. After 8 h incubation, the supernatants were removed, and the infected cells were washed three times with PBS. Bacteria were released from infected macrophages by treatment with 1% Triton X-100 for 10 min. The total bacterial RNA was isolated, and the mRNA levels of z0254, z0264, z0266, z0267, *katG*, *katE*, *katP*, *ahpC* and *katN* were quantified by qPCR.

### The primary peritoneal macrophages isolation

Thioglycolate-elicited primary peritoneal macrophages (Peritoneal MΦ) were harvested as described before [74]. Briefly, BALB/c mice were intraperitoneally injected with 4% Brewer’s thioglycolate medium (Sigma). After 3 days, mice were sacrificed by cervical dislocation, and cells were isolated by flushing the peritoneal cavity with 50 ml PBS per mouse. Cells were seeded in 24-well plates, and non-adherent cells were removed by washing with DMEM. The adherent peritoneal macrophages were used for subsequent experiments.

### Ethics statement

The animal procedures were approved by Shanghai Jiao Tong University School of Medicine, and this study was carried out in strict accordance with the National Research Council Guide for Care and Use of Laboratory Animals [SYXK (Shanghai 2007–0025)]. All surgery was performed under sodium pentobarbital anesthesia, and all efforts were made to minimize suffering.

### Animal experiments

Five-week-old BALB/c mice were used for all animal infection experiments. Two days before infection, mice (8/group) were gavaged with 200 μl PBS (200 mg/ml streptomycin) to disrupt the microbiota in intestinal track. Mice were not fed 8 h before intragastric inoculation. EHEC strain EDL933 and its derived mutants were grown to exponential phase (OD_600_ = 1) followed by washing and resuspension at a concentration of 1×10^10^ CFU in 250 μl of PBS. The body weight and survival of mice were recorded every day from day 0 to day 15 after administration. The extent of bacterial colonization was monitored daily for 8 days by quantitation of the strain EDL933 and its derived mutants shed into fecal pellets. For collecting feces, mice of every group were placed into clean, empty cages, allowed to defecate, and the feces were collected and weighed. The fecal material was diluted 1:10 by weight into sterile PBS then homogenized by vortex. Large debris was pelleted by brief centrifugation at 1,750 g, and the supernatants were diluted and plated onto Sorbitol MacConkey Agar (SMAC) plates to determine CFU/g feces.

### Immuno-fluorescence assay

The immune-fluorescence assays were performed as previously described with minor modifications [38]. RAW264.7 cells were grown in a 24-well tissue culture plate on glass cover-slips at 37°C under 5% of CO_2_. Strain EDL933 and its derived mutants were cultured overnight at 37°C in LB broth. Then, RAW264.7 cells were infected by EDL933 and its mutants (MOI = 10). After incubation for 40 min, the cells were fixed with 4% para-formaldehyde (PFA) in PBS for 30 min at room temperature and then washed three times with PBS. The cells were blocked with 3% BSA-PBS for 30 min at 37°C.

The antibodies were diluted in 3% BSA-PBS at the indicated dilutions: mouse-anti-O157 antibody (1:200); rabbit-anti-KatN antibody (1:6000); goat-anti- mouse FITC conjugate (1:300); goat-anti-rabbit rhodanmine red-X conjugate (1:300). The cover-slips were incubated with anti-O157 and anti-KatN antibodies simultaneously overnight at 4°C. After incubation the cover-slips were washed three times with PBS and were incubated with goat-anti-mouse FITC conjugated and goat-anti-rabbit rhodanmine red-X conjugated antibodies away from light at 37°C for 1 h. Cell nuclei were counterstained with 2.5 μg/ml nuclear stain DAPI for 10 min. The cover-slips were washed three times with PBS and then were mounted on glass slides and sealed with Entellan (Merck). Samples were analyzed using a laser-scanning confocal microscope (Leica TCS-NT) as described previously [13].

### HyPer-3 probe-based reactive oxygen species test

The Hyper-3 probe-based reactive oxygen species test were performed as previously described with some modifications [50]. HyPer-3-transfected RAW264.7 cells were plated on glass coverslips (Fisher) overnight at a cell density of 3×10^5^/ml. Cells were washed three times with PBS and then infected with strain EDL933 and its derived mutants (MOI = 10) in DMEM without Phenol Red and FBS at 37°C under 5% of CO_2_. The uninfected cells were used as a negative control. After incubation for 50 min, the DMEM was removed and cells were washed three times with PBS to remove the extracellular bacteria. Cells were analyzed by a laser-scanning confocal microscope (Leica TCS-NT) at excitation wavelengths of 405 and 488 nm. For every sample, the average 488 nm/405 nm ratios of at least ten cells were analyzed.

### Flow cytometry-based reactive oxygen species (ROS) test

The intracellular ROS were estimated using a fluorescent probe DCFH-DA by flow cytometry [51]. Briefly, RAW264.7 cells were grown overnight in a 24-well tissue culture plate at a cell density of 3×10^5^/ml. Cells were washed three times with PBS and then infected with mutant strains (MOI = 10) in DMEM without Phenol Red and FBS at 37°C under 5% of CO_2_. The uninfected cells were used as a negative control. After incubation for 50 min, the DMEM was removed and cells were washed three times with PBS. DCFH-DA (900 μl, diluted in DMEM without Phenol Red and FBS at a final concentration of 10 μM) was added separately to wells and cells were incubated at 37°C under 5% of CO_2_ for 45 min. After removing DCFH-DA solution, cells were washed three times with PBS and were suspended in 200 μl PBS with propidium iodide (PI) (5 μg/ml). PI was used as a counter stain dye for DCFH. Cells were analyzed by the flow cytometer FACScan (Becton Dickinson). The mean fluorescence of 10000 PI-negative cells was calculated using the flow cytometer software FlowJo, version 6.4.2 (FlowJo, USA).

### TEM-1 translocation assay

The translocation assay was performed as previously described with some modifications [42]. RAW264.7 cells were seeded at 3×10^4^ cells/ml on glass coverslips (Fisher) overnight in minimal essential medium (MEM) with 10% fetal bovine serum (FBS). EHEC strain EDL933 (WT) and ΔT6SS bearing pCX340 or pCX-*katN* were grown overnight in LB broth with 10 μg/ml tetracycline at 37°C, subcultured 1:100 in LB broth with 10 μg/ml tetracycline and 0.8 mM IPTG, followed by growing at 37°C for 2 h. RAW264.7 cells were washed three times with Hanks’ balanced salt solution (HBSS) and infected with 3×10^6^ bacteria (MOI = 100) in MEM medium containing 1% FBS, 10 μg/ml tetracycline and 0.8 mM IPTG for 3 h. Cells were washed three times with HBSS and then incubated with 2 μM CCF2-AM (Invitrogen) for 60 min. Cells were washed three times with HBSS to remove CCF2-AM and then analyzed by a laser-scanning confocal microscope (Leica TCS-NT) at excitation wavelengths of 409 and 488 nm (X20 magnification).

### EHEC interaction with bile salts

EHEC survival in bovine bile salts assay was performed as previously described with modifications [56]. Cells from a single clone were grown overnight in LB broth at 37°C, subcultured 1:100 in 6 ml LB broth. Cells were grown to an OD_600_ of 0.3, at which time cultures were supplemented with 0 mg/ml, 25 mg/ml or 50 mg/ml bovine bile salts (Sigma Aldrich) and incubated at 37°C. At 0 h, 3 h, and 6 h post-bile salt treatment, samples were collected for OD_600_ determination and total RNA isolation.

### Statistical analysis

Descriptive statistics and statistical comparison were performed resourcing to the GraphPad Prism, version 5.00 (GraphPad Software, San Diego, CA). Statistical comparison of mean values between two specific groups was carried out using the Student’s t-test, and ANOVA analysis was applied to compare mean values of more than two groups. *, P<0.05; **, P<0.01; ***, P<0.001; ns, Not significant.

## Supporting information

S1 Table Core ORFs of the T6SS in the genome of strain EDL933 and the annotations of their localization and functions. (DOCX)

S2 Table MS analysis of secretory proteins from the wild type strain and T6SS deletion mutant. (XLSX)

S3 Table Strains and plasmids used in this study. (DOCX)

S4 Table Primers used in this study. (DOCX)

S1 Fig Comparison of T6SS gene clusters between *E*. *coli* strains and other strains including *V*. *cholerae* and *Salmonella* Typhimurium. **Comparison of T6SS gene clusters between *E*. *coli* strains and other strains including *V*. *cholerae* and *Salmonella* Typhimurium.** Genes are represented as arrows. Translated sequences of the most conserved proteins were aligned to the COG sequences and the hits are represented as colored boxes.
(TIF)

S2 Fig Analysis of the average reads per kilobase per million (RPKM) of T6SS gene cluster and whole genome. **Analysis of the average reads per kilobase per million (RPKM) of T6SS gene cluster and whole genome.** The RNA-seq data of EHEC in the Gene Expression Omnibus database (http://www.ncbi.nlm.nih.gov/geo/, accession number: GSE73969) were analyzed. The average reads per kilobase per million (RPKM) was calculated and shown in the figure.
(TIF)

S3 Fig The transcriptional levels of z0264 (*hcp*) under different cultivation conditions. **The transcriptional levels of z0264 (*hcp-2*) under different cultivation conditions.** The wild type strain EDL933 was cultured to an OD_600_ = 1.0 under different cultivation conditions at 37°C or 25°C. Then, the cells were cultured in different media including LB supplemented with 1%, 2% or 0.5% NaCl, LB broth adjusted to pH of 5.4 or 8.1, DMEM medium containing high glucose (4.5 g/L, DMEM H) or low glucose (1.1 g/L, DMEM L). The relative expression levels of z0264 from different samples were analyzed by qPCR. 16S rRNA was used as an internal control. Three biological repeats were performed.
(TIF)

S4 Fig The growth curves of the wild type, T6SS mutant and *katN* mutant strains. **The growth curves of the wild type strain and its derived mutants.** Cells from a single clone were grown overnight in LB broth at 37°C. For growth curve analysis, overnight cultures were inoculated into 50 ml fresh LB medium at an initial OD_600_ of 0.1 at 37°C, and samples were collected hourly for OD_600_ determination.
(TIF)

S5 Fig The isolation of the secreted proteins from EHEC strain EDL933 and the deletion mutant of T6SS. **The isolation of the secreted proteins from EHEC strain EDL933 and the deletion mutant of T6SS.** (A) The SDS-PAGE of the secreted proteins from the WT EHEC strain EDL933 (WT) and the deletion mutant of T6SS (ΔT6SS). (B) The quality assessment of the secretory proteins by Western blot. The secretory proteins were analyzed by Western blot using the cytoplasmic protein RpoA antibody.
(TIF)

S6 Fig Conservation analysis of Z1921 (KatN) of EHEC through sequence alignment. **Conservation analysis of Z1921 (KatN) by sequence alignment.** KatN from EHEC, *Salmonella* Typhimurium, *Citrobacter rodentium*, *Pseudomonas aeruginosa*, *Klebsiella pneumonia*, *Vibrio parahaemolyticus*, *Lactobacillus plantarum*, *Thermus thermophiles*, and *Pyrobaculum calidifontis* were aligned by BioEdit.
(TIF)

S7 Fig The KatN-Bla fusion protein levels at WT and ΔT6SS were comparable. **The KatN-Bla fusion protein levels at WT and ΔT6SS were comparable.** WT and ΔT6SS harboring plasmid pCX340 or pCX-*katN* were cultured to an OD_600_ = 0.3 in LB broth with 10 μg/ml tetracycline at 37°C, and then IPTG was added at a final concentration of 0.5 mM, followed by cultivation to an OD_600_ = 0.8. Total protein samples from each strains were separated on 12% SDS-PAGE, followed by Western blot. Anti-KatN indicated the KatN or KatN-Bla fusion protein. We used purified KatN to indicate the different electrophoretic mobility of KatN and KatN-Bla fusion protein. Anti-RpoA was used as a loading control.
(TIF)

S8 Fig Quantification of blue *vs* total cells in TEM-1 translocation assay. **Quantification of blue *vs* total cells in TEM-1 translocation assay**. One hundred cells were counted to calculate the percentages of blue cells in the RAW264.7 infected by WT-pCX340, WT-pCX*-katN* or ΔT6SS-pCX-*katN*. All the experiments were performed in duplicates.
(TIF)

S9 Fig The secretion of catalase KatN is dependent on z0254 in EHEC. **The secretion of catalase KatN is dependent on z0254 in EHEC.** The wild type EHEC strain EDL933 (WT), ΔT6SS, Δz0254 and Δz0254 complementation strain (Δz0254-c) were cultured to an OD_600_ = 1.0 in LB broth at 37°C. The cytoplasm (CP) and supernatant fractions (SP) of the cultures were analyzed by Western blot using anti-KatN and anti-RpoA antibodies. RpoA was used as an internal control. Three biological repeats were performed.
(TIF)

S10 Fig The secretion of KatN does not rely on T3SS in EHEC. **The secretion of KatN does not rely on T3SS in EHEC.** The WT and ΔT6SS were cultured to an OD_600_ = 1.0 in LB broth at 37°C. The pellet and supernatant fractions of the cultures were analyzed by Western blot using anti-KatN and anti-RpoA monoclonal antibodies. RpoA was used as an internal control. Three biological repeats were performed.
(TIF)

S11 Fig The purification and catalase activity of KatN. **The purification and catalase activity of KatN.** (A) The SDS-PAGE of the purified KatN. KatN was overexpressed and purified as described in “Materials and Methods”. KatN (1 μg) was resolved on 10% SDS-PAGE and stained with Coomassie blue. (B) The catalase activity of KatN. The specific activity of KatN was determined by the catalase assay Kit (Beyotime) according to the instruction. The bovine liver catalase (Sigma) was used as a positive control. The specific activity of KatN was determined as 268.3 U/mg protein.
(TIF)

S12 Fig The catalases KatE, KatG, KatP and AhpC are not secreted *via* T6SS. **The catalases KatE, KatG, KatP and AhpC are not secreted by T6SS.** The wild type strain EDL933 or ΔT6SS harboring pQE80 expressing *katE* (A), *katG* (B), *katP* (C), or *ahpC* (D) with a His-tag sequence fusion at the C-termini were cultured to an OD_600_ = 1.0 in LB broth at 37°C. The cytoplasm (CP) and supernatant fractions (SP) of the cultures were analyzed by Western blot using anti-His tag and anti-RpoA antibodies. RpoA was used as an internal control. Three biological repeats were performed.
(TIF)

S13 Fig The transcription of *katN*, *katE*, *katG*, *katP* and *ahpC in vivo*. **The relative transcriptional levels of *katN*, *katE*, *katG*, *ahpC* and *katP in vivo*.** The wild type strain EDL933 cells were used to infect RAW264.7 cells followed by isolation of the intracellular bacteria, total RNA purification and qPCR analysis. The transcriptional levels of these catalases genes *in vivo* were compared with these of bacteria grown in DMEM medium *in vitro*. 16S rRNA was used as an internal control, and the expression levels of catalases genes *in vitro* were set as 1. Three biological repeats were performed.
(TIF)

S14 Fig KatN decreases the ROS level of macrophage cells. **KatN decreases the ROS level of macrophage cells.** Macrophage RAW264.7 cells were infected with Δ*hns*, Δ*hns*/Δ*katN* or Δ*hns*/Δ*katN-c* (Δ*hns*/Δ*katN* bearing pACYC184-*katN*) at a MOI of 10. After 40 min, DCFH-DA was added, and cells were incubated for another 50 min before the flow cytometer analysis. Propidium iodide (PI) was used as a counter stain dye, and 10000 PI-negative cells were analyzed. ROS levels were shown as the average fluorescence intensities. At least three biological repeats were performed.
(TIF)

S15 Fig T6SS is involved in the survival of EHEC in bile salts. **T6SS is involved in the survival of EHEC in bile salts.** (A) Bile salts promote the transcriptional levels of T6SS genes of EHEC. Total RNA samples were isolated from EHEC cultured in LB broth supplemented with different concentrations of bovine bile salts at different time points at 37°C followed by qPCR analysis. The 16S rRNA was used as internal standard. Error bars represented SD from at least three independent experiments. (B) T6SS contributes to the growth of EHEC at the presence of bile salts. The overnight cultures were inoculated into 6 ml fresh LB broth supplemented with different concentrations of bovine bile salts at an initial OD_600_ of 0.3 at 37°C, and samples were collected at different time points for OD_600_ determination.
(TIF)

S16 Fig T6SS is not involved in the transcription and secretion of Shiga toxin. **T6SS is not involved in the transcription and secretion of Shiga toxin.** (A) T6SS is not involved in the transcription of Shiga toxins. Total RNA was isolated from WT and ΔT6SS at logarithmic phase (OD_600_ = 0.8) in LB broth at 37°C followed by the real-time qPCR analysis. The 16S rRNA was used as internal standard. (B) The absence of T6SS does not disturb the secretion of Shiga toxin. The WT and ΔT6SS bearing pQE80-stx2A with a His-tag sequence fusion at the C-termini were cultured to an OD_600_ = 1.0 in LB broth at 37°C. The pellet and supernatant fractions of the cultures were analyzed by Western blot using anti-His tag and anti-RpoA monoclonal antibodies. RpoA was used as an internal control. Three biological repeats were performed.
(TIF)

S17 Fig The distribution correlation between KatN and T6SS. **The distribution correlation between KatN and the T6SS.** The amino acids sequence of KatN of EHEC was used to identify an additional 129 homologues by using BLASTP searches against the completely sequenced bacterial genomes with strict cutoffs (e-value less or equal to 0.0001 and identity higher or equal to 50%). Finally, 100 KatN homologues (76.9%) were determined to co-distribute with T6SS. In contrast, only 30 KatN homologues (23.1%) were inferred without co-presence of the T6SS gene cluster in the cognate bacterial genomes. Meanwhile, 821 bacterial genomes harboring T6SS gene clusters available in the archived SecReT6 database did not show the presence of KatN homologues.
(TIF)
