# Supplementary material for: Type VI secretion system contributes to Enterohemorrhagic Escherichia coli virulence by secreting catalase against host reactive oxygen species (ROS)
Source: PLoS Pathog. 2017 Mar 13;13(3):e1006246. doi: 10.1371/journal.ppat.1006246 (PMC5363993; doi:10.1371/journal.ppat.1006246)
Supplement: S1 Table — (DOCX) [file ppat.1006246.s001.docx]

**S1 Table. The core T6SS ORFs in the genome of EHEC strain EDL933.**

| **ORFs** | **COG** | **Annotation** | **Localization** | **Putative function** |
| --- | --- | --- | --- | --- |
| z0248 | COG3157 | Hcp family protein | OM | T6SS effector |
| z0249 | COG3515 | ImpA-related N-terminal | IM | T6SS essential |
| z0250 | COG3523 | IcmF/VasK like | IM | T6SS essential |
| z0251 | COG3515 | ImpA-related N-terminal | IM | T6SS essential |
| z0252 | COG3515 | ImpA-related N-terminal | IM | T6SS essential |
| z0253 |  | VasH like | PM | T6SS essential |
| z0254 | COG0542 | ClpV | CM | T6SS ATPase |
| z0255 | COG3455 | DotU like | IM | Cell attachment site |
| z0256 | COG3522 | VgrE like | CM | T6SS protein VCA0114 family |
| z0257 | COG3521 | SciN like | OM | T6SS lipoprotein |
| z0258 | COG3456 | FHA protein | IM | T6SS FHA domain protein |
| z0260 | COG3519 | VasA like | IM | T6SS essential |
| z0261 | COG3518 | gp25 like protein | CM | T6SS related |
| z0264 | COG3157 | Hcp family protein | OM | T6SS effector |
| z0266 | COG3157 | Hcp family protein | OM | T6SS effector |
| z0267 | COG3501 | VgrG like | OM | T6SS Vgr family protein |
| z0268 | COG3209 | Rhs | CM | Recombination hot spot elements |
| z0707 | COG3501 | VgrG like | OM | T6SS Vgr family protein |
| z2262 | COG3501 | VgrE like | OM | T6SS Vgr family protein |
