# Supplementary material for: Type VI secretion system contributes to Enterohemorrhagic Escherichia coli virulence by secreting catalase against host reactive oxygen species (ROS)
Source: PLoS Pathog. 2017 Mar 13;13(3):e1006246. doi: 10.1371/journal.ppat.1006246 (PMC5363993; doi:10.1371/journal.ppat.1006246)
Supplement: S3 Table — (DOCX) [file ppat.1006246.s003.docx]

**S3 Table. Strains and plasmids used in this study**

| **Strains/plasmids** | **Relevant characteristics** | **Sources** |
| --- | --- | --- |
| **Strains** |  |  |
| EDL933 | EHEC O157: H7 isolated in 1982 from polluted food, producing Stx | Lab stock |
| ΔT6SS | T6SS gene cluster deletion mutant derived from EHEC strain EDL933 | This study |
| ΔT3SS | *escN* deletion mutant derived from EHEC strain EDL933 | Lab stock |
| Δz0254 | z0254 (*clpV*) gene deletion mutant derived from strain EDL933 | This study |
| Δ*rpoS* | z4049 (*rpoS*) deletion mutant derived from strain EDL933 | This study |
| Δ*oxyR* | z5519 (*oxyR*) deletion mutant derived from strain EDL933 | This study |
| Δ*hns* | z2013 (*hns*) deletion mutant derived from strain EDL933 | This study |
| Δ*hns*Δ*katN* | z2013 (*hns*) and *katN* double deletion mutant derived from strain EDL933 | This study |
| MG1655 | *E. coli* K-12 strain MG1655 | Lab stock |
| PAO1 | *Pseudomonas aeruginosa* strain PAO1 | Mougous J |
| Δ*retS* | *retS* deletion mutant derived from strain PAO1 | Mougous J |
| Δ*ppkA* | *ppkA* deletion mutant derived from strain PAO1 | Mougous J |
| *A. baylyi* | *Acinetobacter baylyi* strain ADP1 | Mougous J |
| **Plasmids** |  |  |
| pQE80YX1 | The vector with *lacI*q repressor gene for expressing C-terminal RGS-6 His-tagged protein | Lab stock |
| pQE80-z5583 | pQE80YX1 harboring z5583 at *Afe* I and *Fse* I sites | This study |
| pQE80-z0873 | pQE80YX1 harboring z0873 at *Afe* I and *Fse* I sites | This study |
| pQE80-*katG* | pQE80YX1 harboring *katG* (z5497) at *Afe* I and *Fse* I sites | This study |
| pQE80-*katE* | pQE80YX1 harboring *katE* (z2761) at *Afe* I and *Fse* I sites | This study |
| pQE80-*katP* | pQE80YX1 harboring *katP* at *Afe* I and *Fse* I sites | This study |
| pQE80-*ahpC* | pQE80YX1 harboring *ahpC* at *Afe* I and *Fse* I sites | This study |
| pQE80-z0264 | pQE80YX1 harboring z0264 (*hcp-2*) at the *Afe* I and *Fse* I sites | This study |
| pQE80-*katN* | pQE80YX1 harboring z1921 at *Afe* I and *Fse* I sites | This work |
| pQE80-z0254*-gfp* | pQE80YX1 harboring *clpV* and *gfp* at *Afe*I and *Fse* I sites | This study |
| pCX340 | pBR322 derivative vector used to fuse putative effectors to the mature form of β-lactamase | Lab stock |
| pCX-*katN* | pCX340 harboring z1921 (*katN*) at *Nde* I and *EcoR* I sites | This study |
| pACYC184 | *E. coli* plasmid cloning vector containing the p15A origin of replication | Lab stock |
| pACYC184-z0264 | pACYC184 vector harboring C-6XHis-z0264 (*hcp-2*) and its promoter at *Hind* III and *BamH* I sites | This study |
| pACYC184-*katN* | pACYC184 harboring C-6XHis-z1921 and its promoter at *Hind* III and *BamH* I sites | This study |
| pACYC184-*hns* | pACYC184 harboring *hns* (z2013)and its promoter at *Hind* III and *BamH* I sites | This study |
