# Supplementary material for: Type VI secretion system contributes to Enterohemorrhagic Escherichia coli virulence by secreting catalase against host reactive oxygen species (ROS)
Source: PLoS Pathog. 2017 Mar 13;13(3):e1006246. doi: 10.1371/journal.ppat.1006246 (PMC5363993; doi:10.1371/journal.ppat.1006246)
Supplement: S4 Table — (DOCX) [file ppat.1006246.s004.docx]

**S4 Table. Primers used in this study.**

| **Primers** | **Sequences(5’ – 3’)** |
| --- | --- |
| T6SS KO-F | GTTTCCACATTCTCTGGTATCCCGGACAGGCAAAAGCCTCTGACTTGTAAGTGTAGGCTGGAGCTGCTTC |
| T6SS KO-R | TCTAACACCCAATGAAGACTGTGCTCGATCAGCCAGTGCGCTCTGATAGCCATATGAATATCCTCCTTA |
| T6SS KO CHK-F | AGCGCTTGATGGAGATGAGT |
| T6SS KO CHK-R | GGCTGGCATCTTCATTCATT |
| z0254-KO-F | ACGCAGGACATCCTGCACCAGCTAAACAAATTACTTCGATAAGGACGTTTGTGTAGGCTGGAGCTGCTT |
| z0254-KO-R | CCACTGCCGACAACCGAATCCGGGCAATCGCCGTGTTAGCCGCCATTTTCCATATGAATATCCTCCTTAGTT |
| z0254-KO-CHK-F | TTCCCGCTGTTACACCAGG |
| z0254-KO-CHK-R | GCCATTGACGATGCTTCTG |
| katN-KO-F | AATTCATGGTCAGATCTGAAATTGATGGAGTTGAAGCCAAAAAATAATTCTTTTGAGGAGGCATTGTGTAGGCTGGAGCTGCTTC |
| katN-KO-R | CTAGAGCTTAGGATATCTCAGCGGCAATTAACTTATGAGGGATAGCTAAATACCAGAAGATAACATATGAATATCCTCCTTAGTT |
| katN-KO-CHK-F | ACACATCTCTTCTCGCAGCA |
| katN-KO-CHK-R | TCGGGTGTTGCTGAACCGT |
| rpoS-KO-F | AGGCTTTTGCTTGAATGTTCCGTCAAGGGATCACGGGTAGGAGCCACCTTGTGTAGGCTGGAGCTGCTTC |
| rpoS-KO-R | AAGAAAAAGGCCAGCCTCGCTTGAGACTGGCCTTTCTGACAGATGCTTACCATATGAATATCCTCCTTA |
| rpoS-KO-CHK-F | ATCGGCGGAACCAGGCTTTT |
| rpoS-KO-CHK-R | GCAAGATGATGAACACATAGG |
| oxyR-KO-F | TTGCTATTCTACCTATCGCCATGAACTATCGTGGCGATGGAGGATGGATAGTGTAGGCTGGAGCTGCTTC |
| oxyR-KO-R | TTAACTACCCGACGATGGCGGAAGCCTATCGGGTAGCTGCGTTAAACGGTCATATGAATATCCTCCTTA |
| oxyR-KO-CHK-F | CAGTCAGAATGCTTGATAGG |
| oxyR-KO-CHK-R | ACACTATTGAGTACTTCGTCA |
| hns-KO-F | TCTATTATTACCTCAACAAACCACCCCAATATAAGTTTGAGATTACTACAGTGTAGGCTGGAGCTGCTTC |
| hns-KO-R | TAAAAAATCCCGCCGATGGCGGGATTTTAAGCAAGTGCAATCTACAAAAGACATATGAATATCCTCCTTAGTTC |
| hns-KO-CHK-F | GTACAAATAGGGCTATATGCC |
| hns-KO-CHK-R | GAAGAAGAGATGGGCATTAAG |
| pQE80-katN-F | ATGTTTAGACACGTGAAAAAA |
| pQE80-katN-R | CGGGGCCGGCCTGTTTTTACTATTTATCTTTGTTTG |
| pQE80-z5583-F | ATGCAACAACGTCGTCCAGT |
| pQE80-z5583-R | CGGGGCCGGCCTATGGCGGAAGTGGCGCATA |
| pQE80-z0873-F | ATGGCTGATACAAAAGCAAAAC |
| pQE80-z0873-R | CGGGGCCGGCCTATGTTTAACATCTGATTTAAAGTC |
| pQE80-katP-F | ATGATAAAAAAAACTCTTCCTGTT |
| pQE80-katP-R | CGGGGCCGGCCTTTTATTGTTTAAATCAAACCGATC |
| pQE80-ahpC-F | ATGTCCTTGATTAACACCAAAA |
| pQE80-ahpC-R | CGGGGCCGGCCTGATTTTACCAACCAGGTCCA |
| PQE80-katG-F | GAGGAGAAATTAAGCATGAGCACGTCAGACGATATC |
| pQE80-katG-R | GTGATGCGATCCTCTGGCCGGCCTCAGCAGGTCGAAACGGTCG |
| pQE80-katE-F | GAGGAGAAATTAAGCATGTCGCAACAAAACGAAAAGA |
| pQE80-katE-R | GTGATGCGATCCTCTGGCCGGCCTCGCCGGAATTTTGTCAATCTT |
| pQE80-z0254-gfp-F1 | TTAAAGAGGAGAAATTAAGCATGATCCAGATTGATCTTCCC |
| pQE80-z0254-gfp-R1 | GTGATGGTGATGGTGATGCGATCCTCTTAAAACCGTTTCATCCTTTGTGA |
| pQE80-z0254-gfp-F2 | TCGCATCACCATCACCATCACATGAGTAAAGGAGAAGAACTTTT |
| pQE80-z0254-gfp-R2 | ATGCGATCCTCTGGCCGGCCTTTATTTGTATAGTTCATCCATGC |
| pQE80-CHK-F | GTCTTCACCTCGAGAAATCA |
| pQE80-CHK-R | CTAGCTTGGATTCTCACCAA |
| katN-NdeI-F | CCCCATATGTTTAGACACGTGAAAAAACTTCA |
| katN-EcoRI-R | CCCGAATTCTCGTGATGGTGATGGTGATGGTTTTTACTATTTATCTTTGTTTGT |
| pCX340-CHK-F | TGTGTGGAATTGTGAGCGGA |
| pCX340-CHK-R | ATACCGCGCCACATAGCAG |
| pACYC-katN-F | CCCAAGCTTCGTGTTACACATCTCTTCTCG |
| pACYC-katN-R | CGCGGATCCTTAGTGATGGTGATGGTGATGGTTTTTACTATTTATCTTTGTTTGT |
| pACYC-z0264-F | CGCAAGCTTTCTATTTAACGCTTGCAGAATAC |
| pACYC-z0264-R | GCGGGATCCTTAGTGATGGTGATGGTGATGTTTATTGCTCACCTGGCCGAT |
| pACYC-hns-F | CGCGGATCCAGCCACAGGCCCTCAATGAT |
| pACYC-hns-R | CCCAAGCTTTTATTGCTTGATCAGGAAATCG |
| pACYC-CHK-F | TTCTAGATTTCAGTGCAATTTATC |
| pACYC-CHK-R | CGGTGATGTCGGCGATATAG |
| katN-RT-F | GATGAAGATGCAGGCCGAAG |
| katN-RT-R | AGTTAATGCTGGGCCTCCTC |
| hns-RT-F | GAACGAGCTGCTGAATAGCC |
| hns-RT-R | CCTTGCTCATCCATTGCTTT |
| katG-RT-F | GATCTGAAAGCCCTGCTGAC |
| katG-RT-R | CCCAGGAGATTTTCTGACCA |
| katE-RT-F | AGAACCACACTGGGCAATTC |
| katE-RT-R | CTTCCCTTCGGCATTAATCA |
| katP-RT-F | CGCCAACCCAGTTCACAATG |
| katP-RT-R | TCAACGGATGCAGGACAGAC |
| ahpC-RT-F | CCCTGACCCGTAACTTCGAC |
| ahpC-RT-R | CAGAGACGGAGCCAGAGTTG |
| z0264-RT-F | GAACGTCAGGCAGTTTCCGT |
| z0264-RT-R | GGCCACGCTATCTGGTGAAA |
| z0266-RT-F | ACGTTACTGTCCCGACCGAT |
| z0266-RT-R | GCTCCTGTTTGCCTTCCACA |
| z0267-RT-F | TGCGGTGGTCTCCTTTCATC |
| z0267-RT-R | GCCATATCGTCAGGTAGGCC |
| z0254-RT-F | GACTGCGTACCGACACATCA |
| z0254-RT-R | GATAATCTCCCGGACCAGCG |
| stx1 A-RT-F | GGATGATCTCAGTGGGCGTT |
| stx1 A-RT-R | ATGCCACGCTTCCCAGAATT |
| stx2 B-RT-F | TACAGTGAAGGTTGACGGGA |
| stx2 B-RT-R | GCACTTCAGCAAATCCGGAG |
| pQE80-stx2A-F | ATGAAGTGTATATTATTTAA |
| pQE80-stx2A-R | TATAGGCCGGCCTTATTTACCCGTTGTATATA |
