# Supplementary figures and images for: Type VI secretion system contributes to Enterohemorrhagic Escherichia coli virulence by secreting catalase against host reactive oxygen species (ROS)
Source: PLoS Pathog. 2017 Mar 13;13(3):e1006246. doi: 10.1371/journal.ppat.1006246 (PMC5363993; doi:10.1371/journal.ppat.1006246)

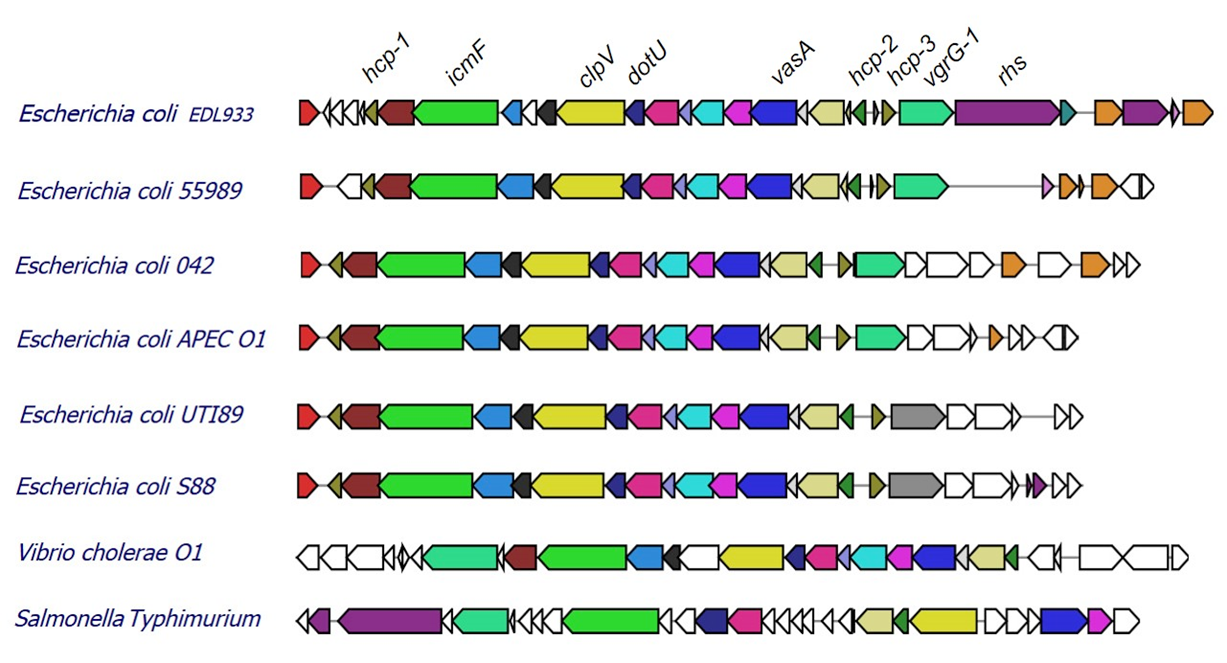

Supplement: S1 Fig — Comparison of T6SS gene clusters between E. coli strains and other strains including V. cholerae and Salmonella Typhimurium. Genes are represented as arrows. Translated sequences of the most conserved proteins were aligned to the COG sequences and the hits are represented as colored boxes. (TIF) [file ppat.1006246.s005.tif]

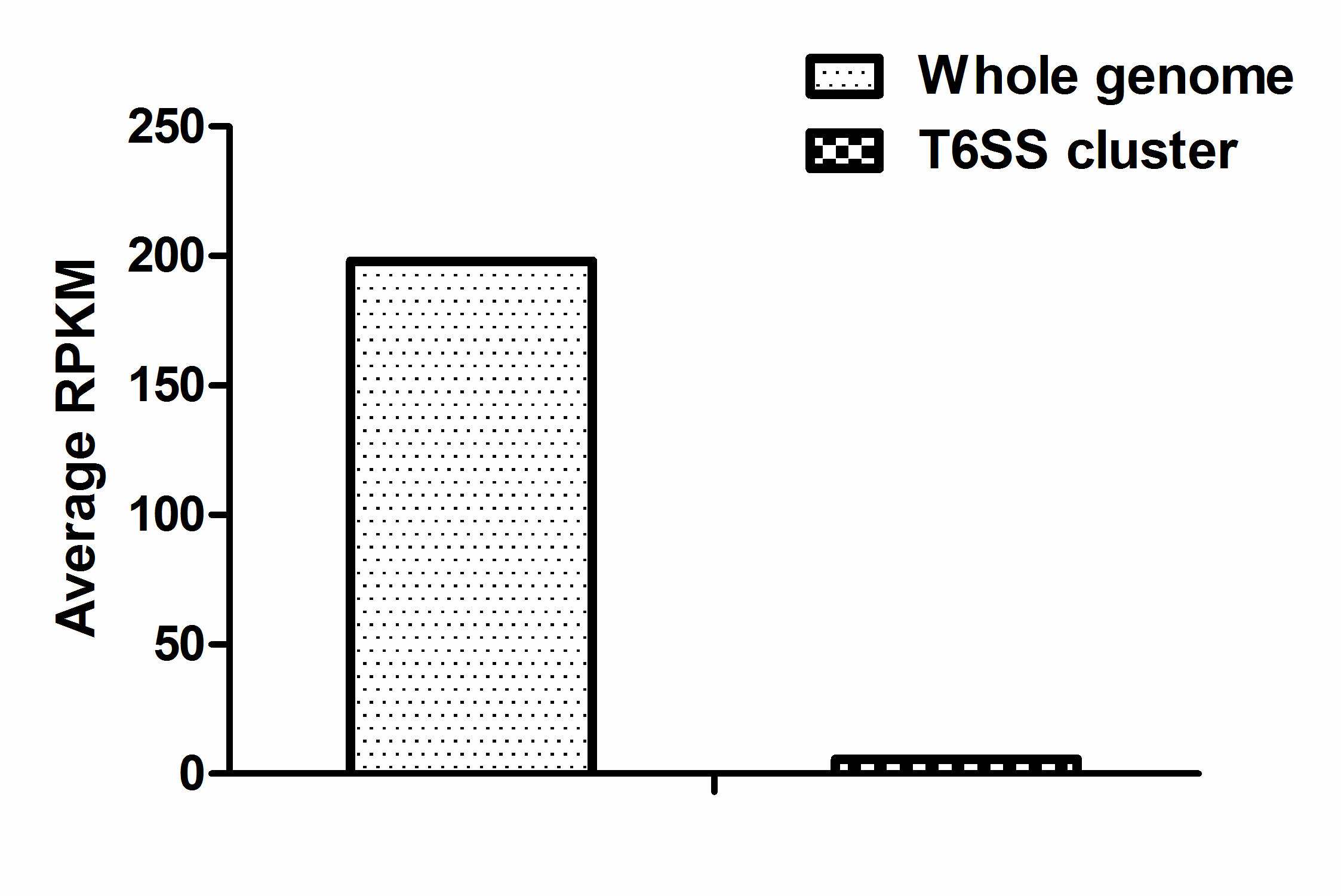

Supplement: S2 Fig — Analysis of the average reads per kilobase per million (RPKM) of T6SS gene cluster and whole genome. The RNA-seq data of EHEC in the Gene Expression Omnibus database (http://www.ncbi.nlm.nih.gov/geo/, accession number: GSE73969) were analyzed. The average reads per kilobase per million (RPKM) was calculated and shown in the figure. (TIF) [file ppat.1006246.s006.tif]

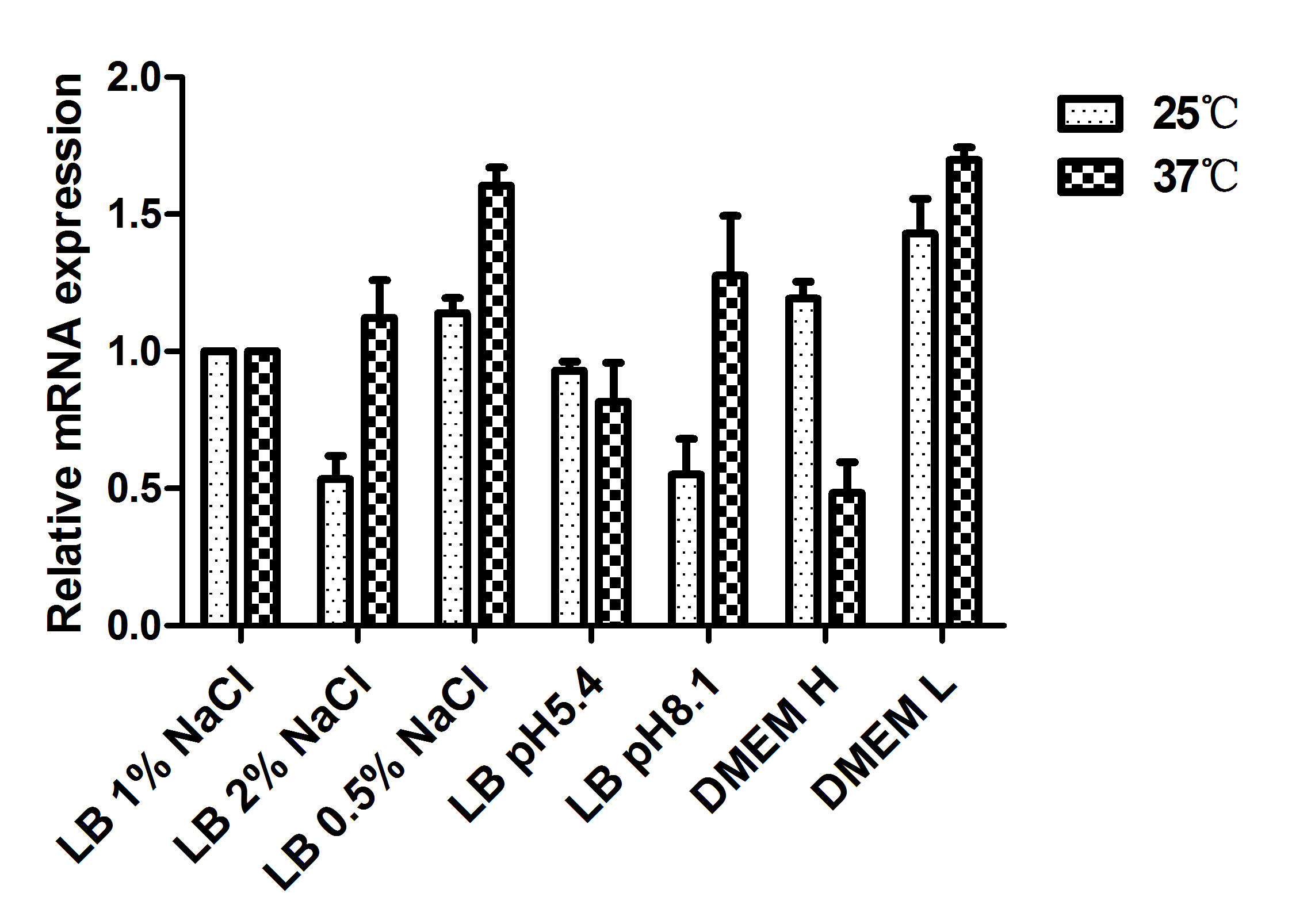

Supplement: S3 Fig — The transcriptional levels of z0264 (hcp-2) under different cultivation conditions. The wild type strain EDL933 was cultured to an OD600 = 1.0 under different cultivation conditions at 37°C or 25°C. Then, the cells were cultured in different media including LB supplemented with 1%, 2% or 0.5% NaCl, LB broth adjusted to pH of 5.4 or 8.1, DMEM medium containing high glucose (4.5 g/L, DMEM H) or low glucose (1.1 g/L, DMEM L). The relative expression levels of z0264 from different samples were analyzed by qPCR. 16S rRNA was used as an internal control. Three biological repeats were performed. (TIF) [file ppat.1006246.s007.tif]

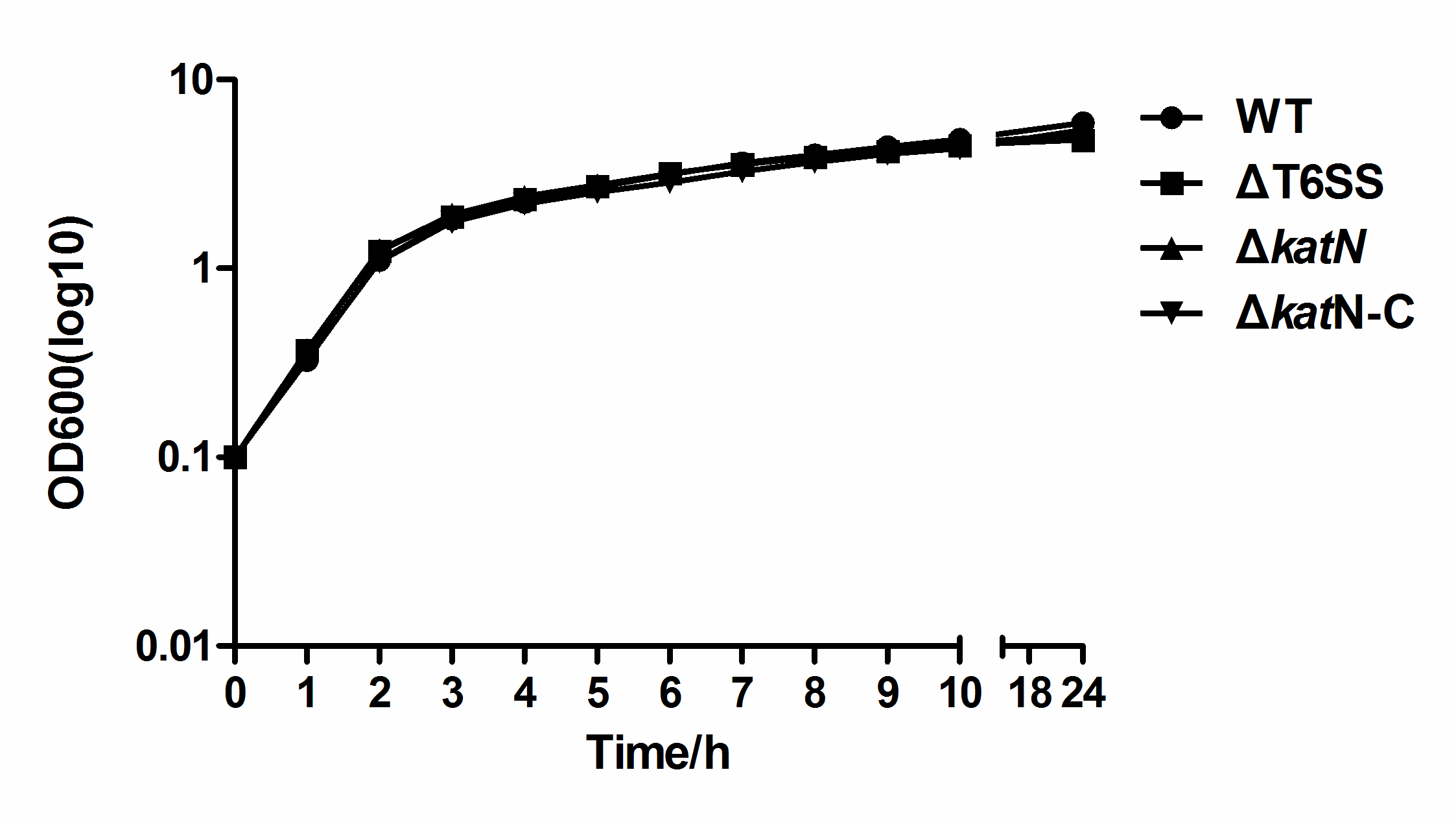

Supplement: S4 Fig — The growth curves of the wild type strain and its derived mutants. Cells from a single clone were grown overnight in LB broth at 37°C. For growth curve analysis, overnight cultures were inoculated into 50 ml fresh LB medium at an initial OD600 of 0.1 at 37°C, and samples were collected hourly for OD600 determination. (TIF) [file ppat.1006246.s008.tif]

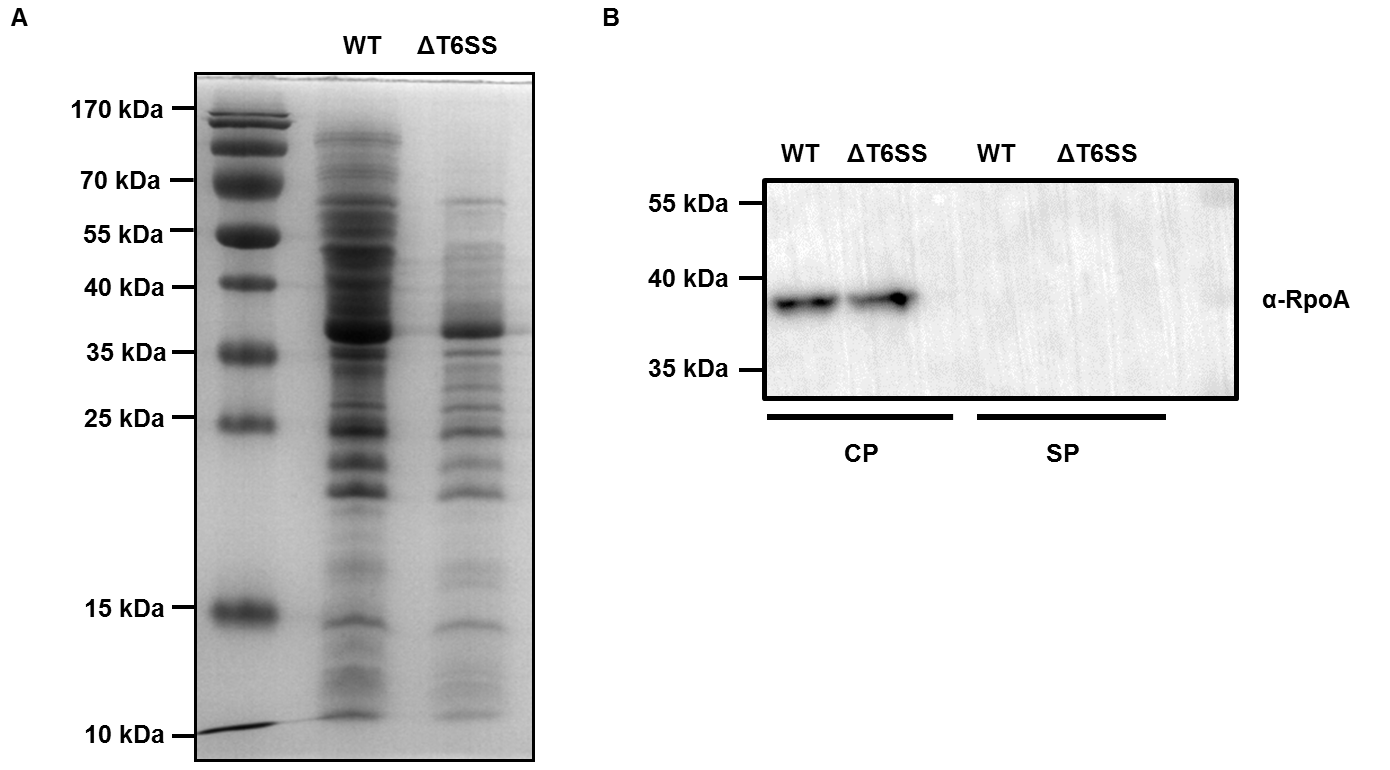

Supplement: S5 Fig — The isolation of the secreted proteins from EHEC strain EDL933 and the deletion mutant of T6SS. (A) The SDS-PAGE of the secreted proteins from the WT EHEC strain EDL933 (WT) and the deletion mutant of T6SS (ΔT6SS). (B) The quality assessment of the secretory proteins by Western blot. The secretory proteins were analyzed by Western blot using the cytoplasmic protein RpoA antibody. (TIF) [file ppat.1006246.s009.tif]

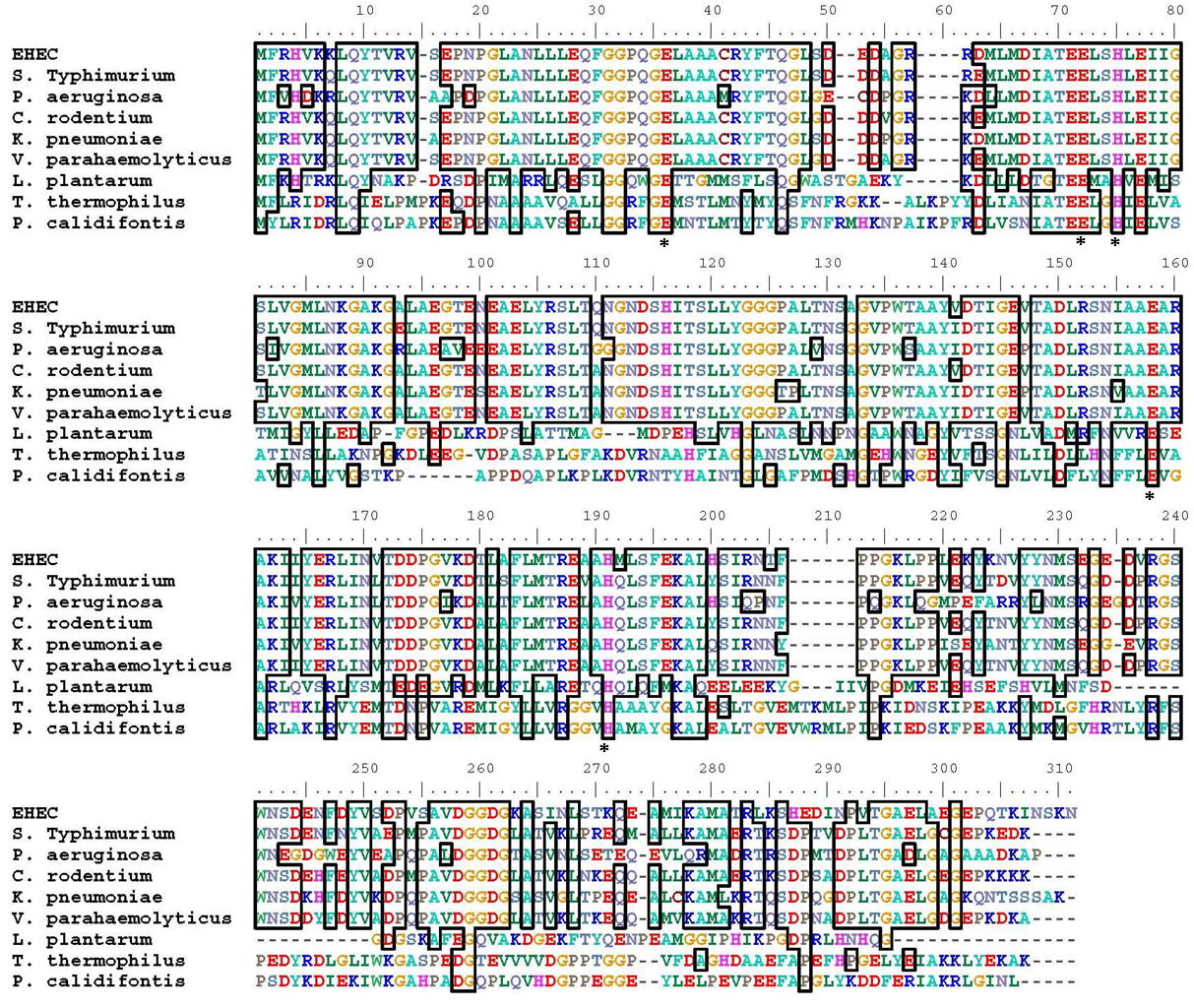

Supplement: S6 Fig — Conservation analysis of Z1921 (KatN) by sequence alignment. KatN from EHEC, Salmonella Typhimurium, Citrobacter rodentium, Pseudomonas aeruginosa, Klebsiella pneumonia, Vibrio parahaemolyticus, Lactobacillus plantarum, Thermus thermophiles, and Pyrobaculum calidifontis were aligned by BioEdit. (TIF) [file ppat.1006246.s010.tif]

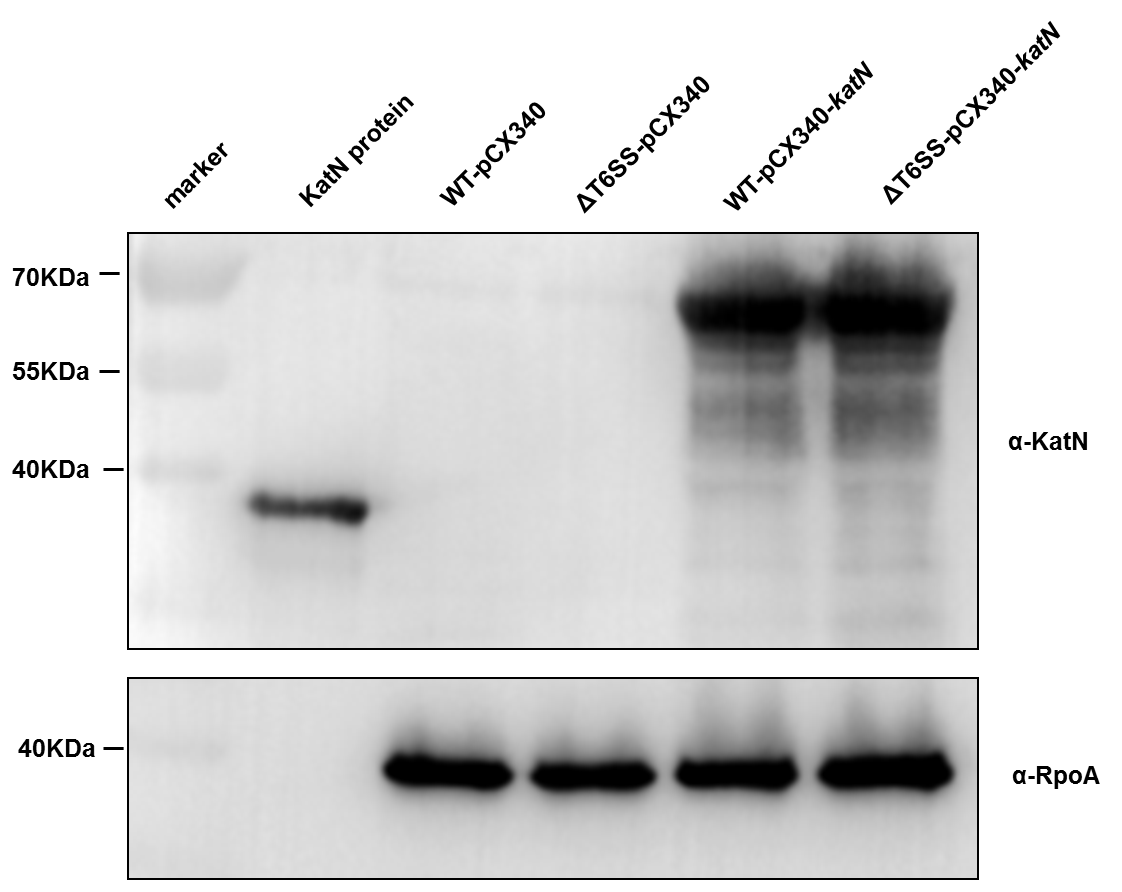

Supplement: S7 Fig — The KatN-Bla fusion protein levels at WT and ΔT6SS were comparable. WT and ΔT6SS harboring plasmid pCX340 or pCX-katN were cultured to an OD600 = 0.3 in LB broth with 10 μg/ml tetracycline at 37°C, and then IPTG was added at a final concentration of 0.5 mM, followed by cultivation to an OD600 = 0.8. Total protein samples from each strains were separated on 12% SDS-PAGE, followed by Western blot. Anti-KatN indicated the KatN or KatN-Bla fusion protein. We used purified KatN to indicate the different electrophoretic mobility of KatN and KatN-Bla fusion protein. Anti-RpoA was used as a loading control. (TIF) [file ppat.1006246.s011.tif]

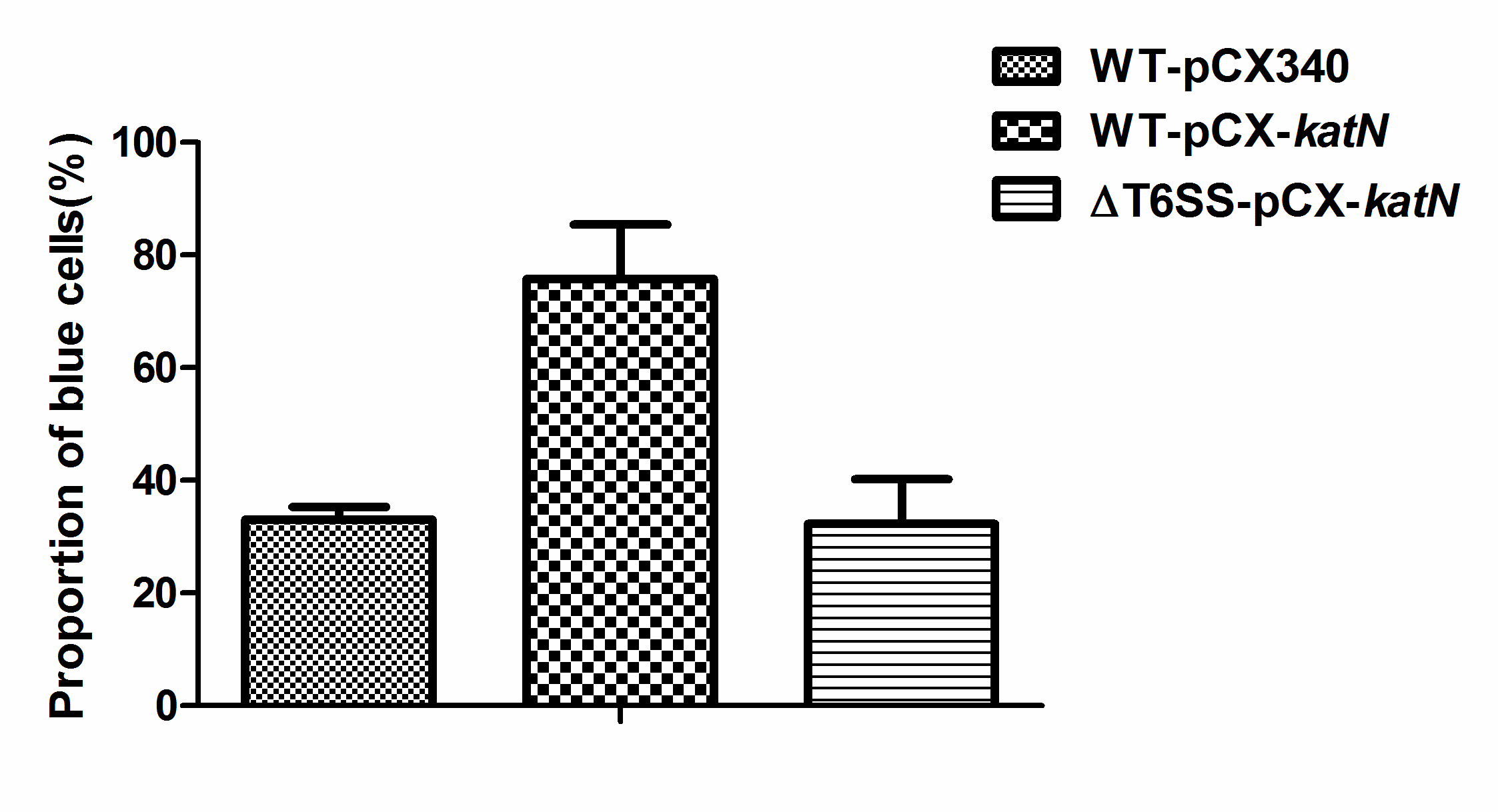

Supplement: S8 Fig — Quantification of blue vs total cells in TEM-1 translocation assay. One hundred cells were counted to calculate the percentages of blue cells in the RAW264.7 infected by WT-pCX340, WT-pCX-katN or ΔT6SS-pCX-katN. All the experiments were performed in duplicates. (TIF) [file ppat.1006246.s012.tif]

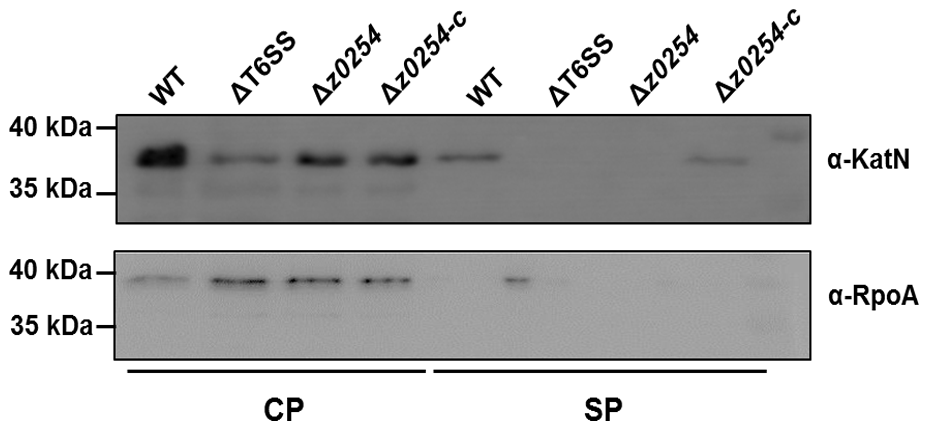

Supplement: S9 Fig — The secretion of catalase KatN is dependent on z0254 in EHEC. The wild type EHEC strain EDL933 (WT), ΔT6SS, Δz0254 and Δz0254 complementation strain (Δz0254-c) were cultured to an OD600 = 1.0 in LB broth at 37°C. The cytoplasm (CP) and supernatant fractions (SP) of the cultures were analyzed by Western blot using anti-KatN and anti-RpoA antibodies. RpoA was used as an internal control. Three biological repeats were performed. (TIF) [file ppat.1006246.s013.tif]

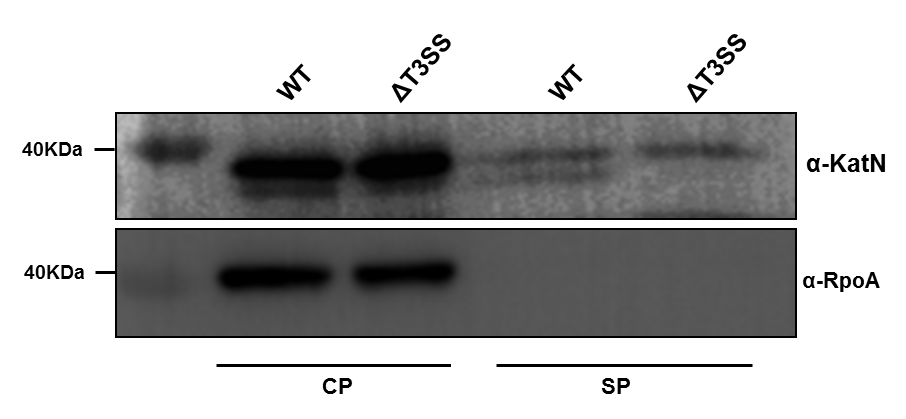

Supplement: S10 Fig — The secretion of KatN does not rely on T3SS in EHEC. The WT and ΔT6SS were cultured to an OD600 = 1.0 in LB broth at 37°C. The pellet and supernatant fractions of the cultures were analyzed by Western blot using anti-KatN and anti-RpoA monoclonal antibodies. RpoA was used as an internal control. Three biological repeats were performed. (TIF) [file ppat.1006246.s014.tif]

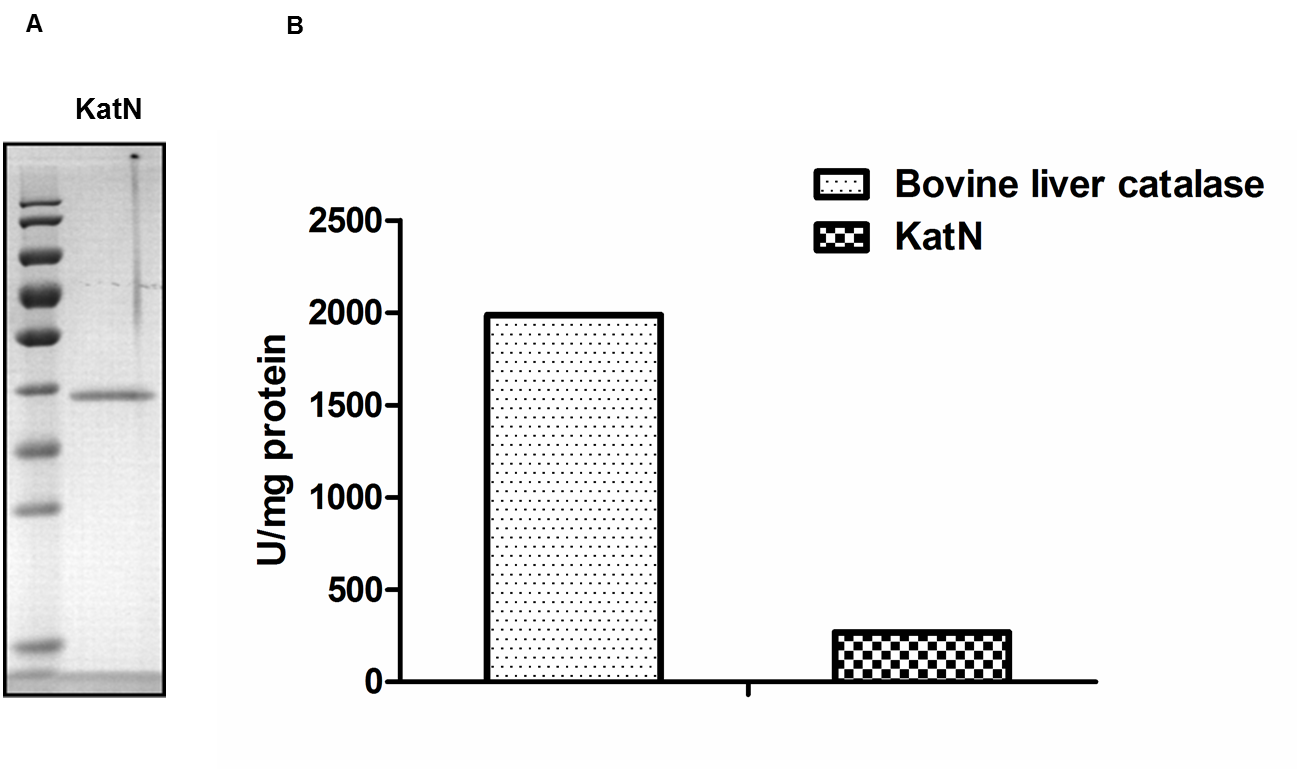

Supplement: S11 Fig — The purification and catalase activity of KatN. (A) The SDS-PAGE of the purified KatN. KatN was overexpressed and purified as described in “Materials and Methods”. KatN (1 μg) was resolved on 10% SDS-PAGE and stained with Coomassie blue. (B) The catalase activity of KatN. The specific activity of KatN was determined by the catalase assay Kit (Beyotime) according to the instruction. The bovine liver catalase (Sigma) was used as a positive control. The specific activity of KatN was determined as 268.3 U/mg protein. (TIF) [file ppat.1006246.s015.tif]

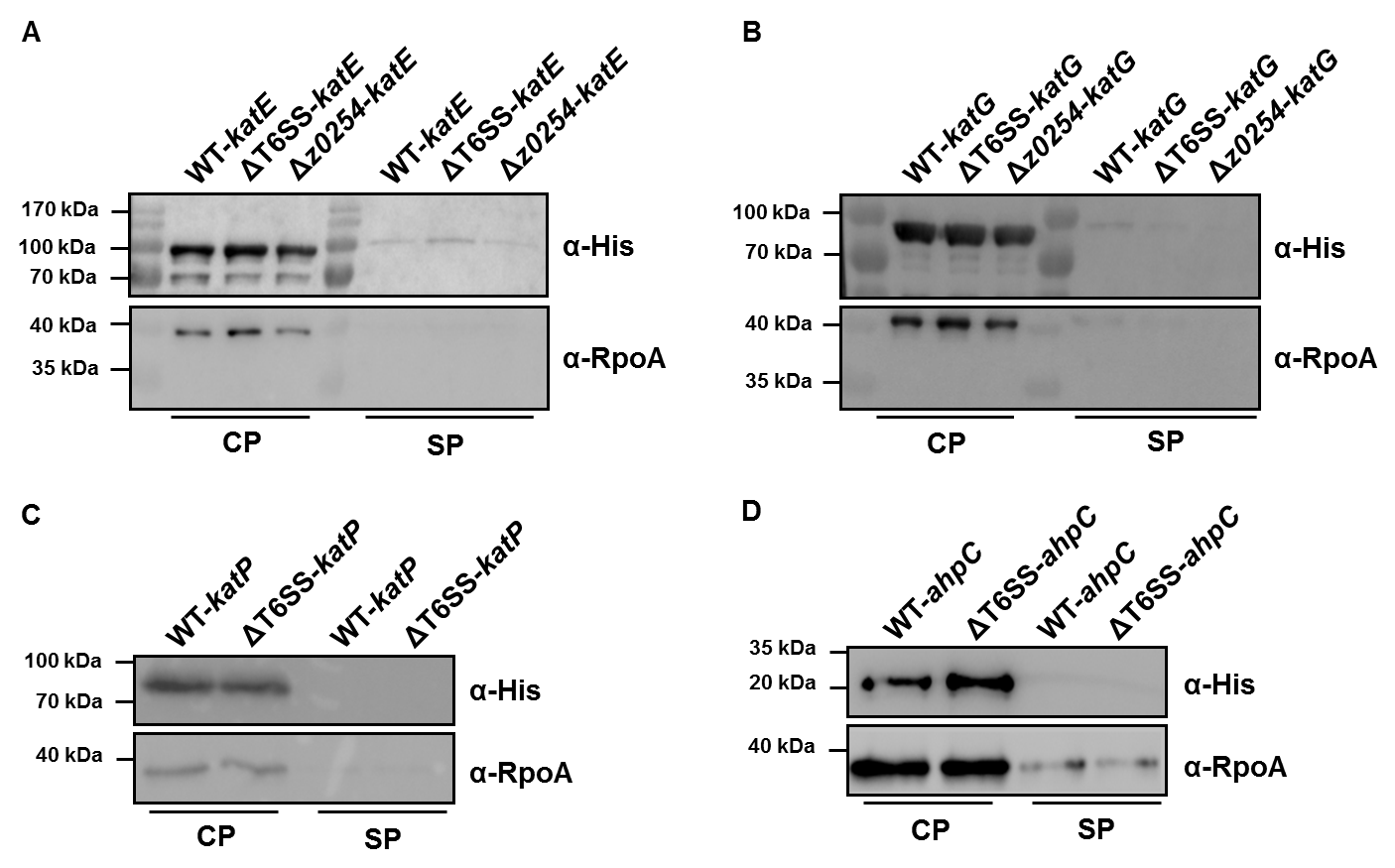

Supplement: S12 Fig — The catalases KatE, KatG, KatP and AhpC are not secreted by T6SS. The wild type strain EDL933 or ΔT6SS harboring pQE80 expressing katE (A), katG (B), katP (C), or ahpC (D) with a His-tag sequence fusion at the C-termini were cultured to an OD600 = 1.0 in LB broth at 37°C. The cytoplasm (CP) and supernatant fractions (SP) of the cultures were analyzed by Western blot using anti-His tag and anti-RpoA antibodies. RpoA was used as an internal control. Three biological repeats were performed. (TIF) [file ppat.1006246.s016.tif]

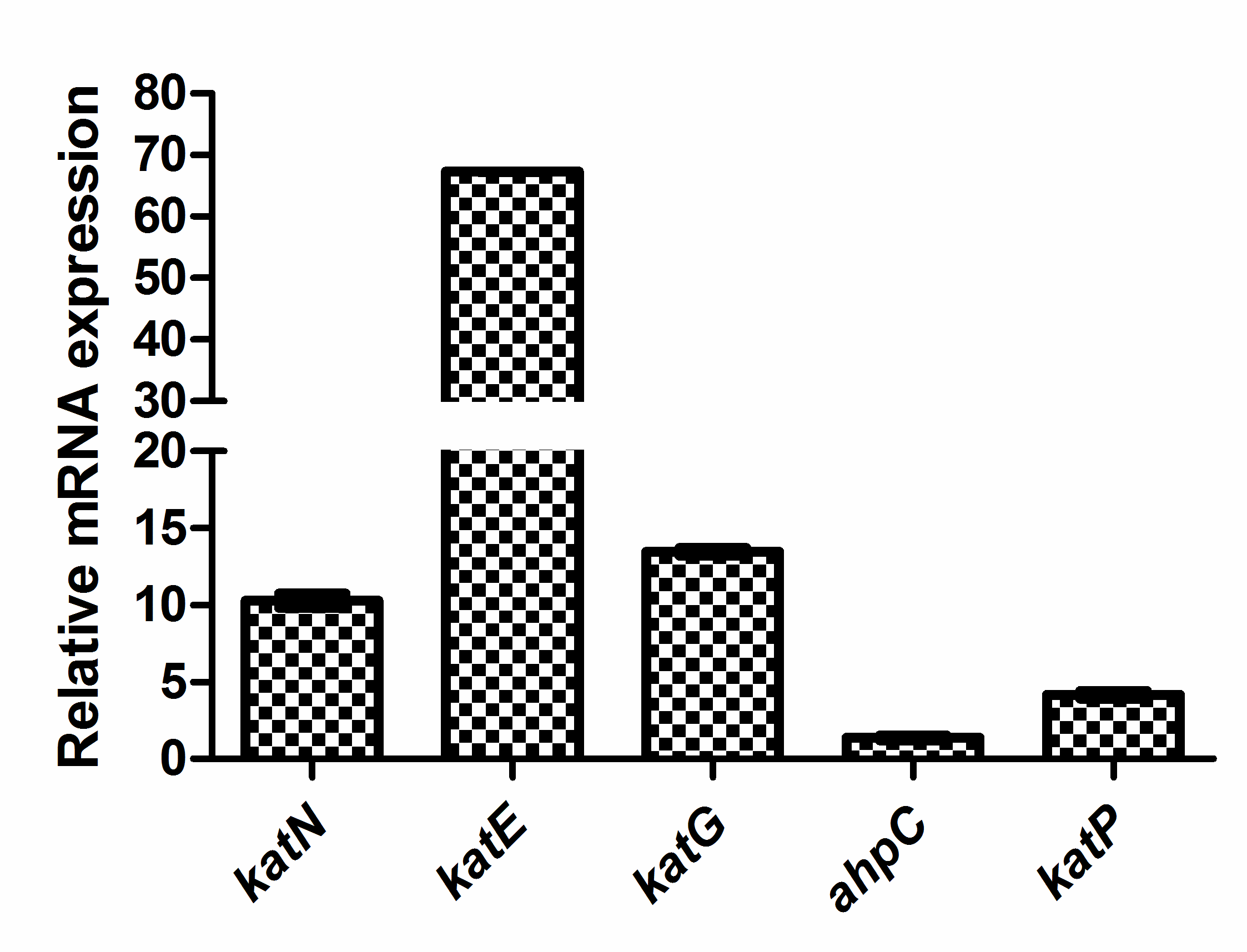

Supplement: S13 Fig — The relative transcriptional levels of katN, katE, katG, ahpC and katP in vivo. The wild type strain EDL933 cells were used to infect RAW264.7 cells followed by isolation of the intracellular bacteria, total RNA purification and qPCR analysis. The transcriptional levels of these catalases genes in vivo were compared with these of bacteria grown in DMEM medium in vitro. 16S rRNA was used as an internal control, and the expression levels of catalases genes in vitro were set as 1. Three biological repeats were performed. (TIF) [file ppat.1006246.s017.tif]

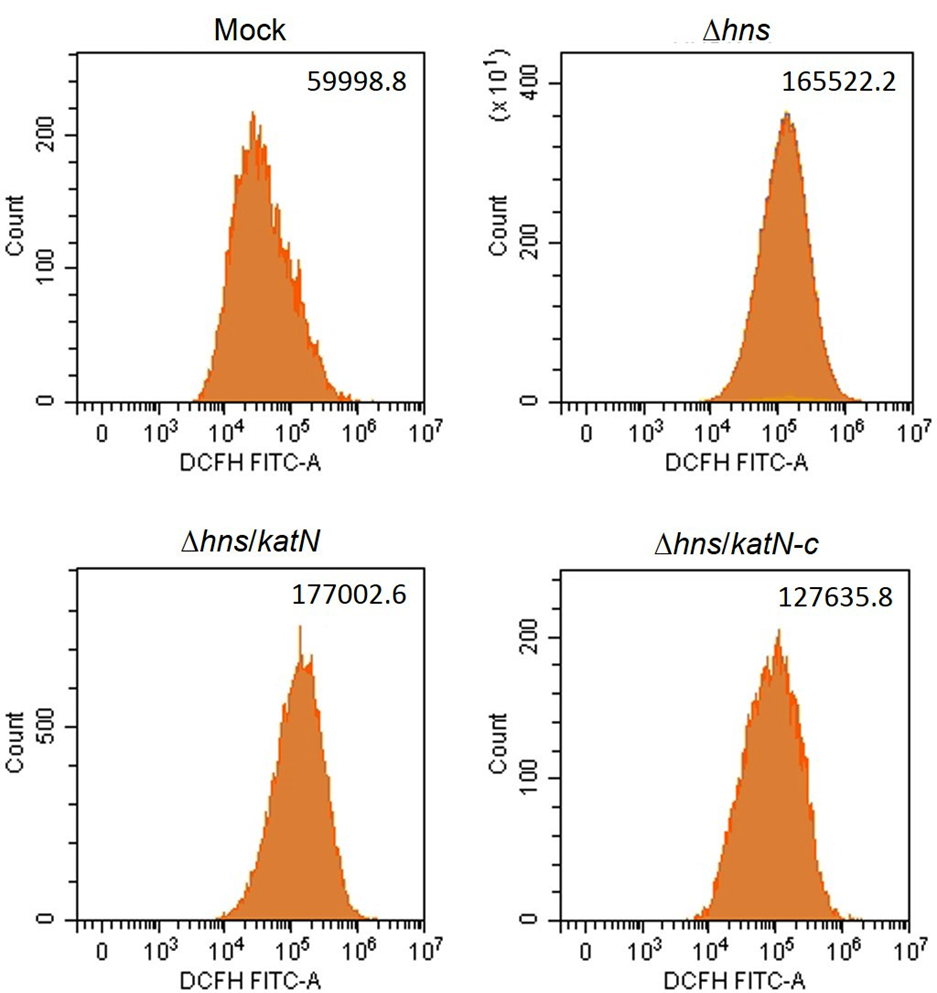

Supplement: S14 Fig — KatN decreases the ROS level of macrophage cells. Macrophage RAW264.7 cells were infected with Δhns, Δhns/ΔkatN or Δhns/ΔkatN-c (Δhns/ΔkatN bearing pACYC184-katN) at a MOI of 10. After 40 min, DCFH-DA was added, and cells were incubated for another 50 min before the flow cytometer analysis. Propidium iodide (PI) was used as a counter stain dye, and 10000 PI-negative cells were analyzed. ROS levels were shown as the average fluorescence intensities. At least three biological repeats were performed. (TIF) [file ppat.1006246.s018.tif]

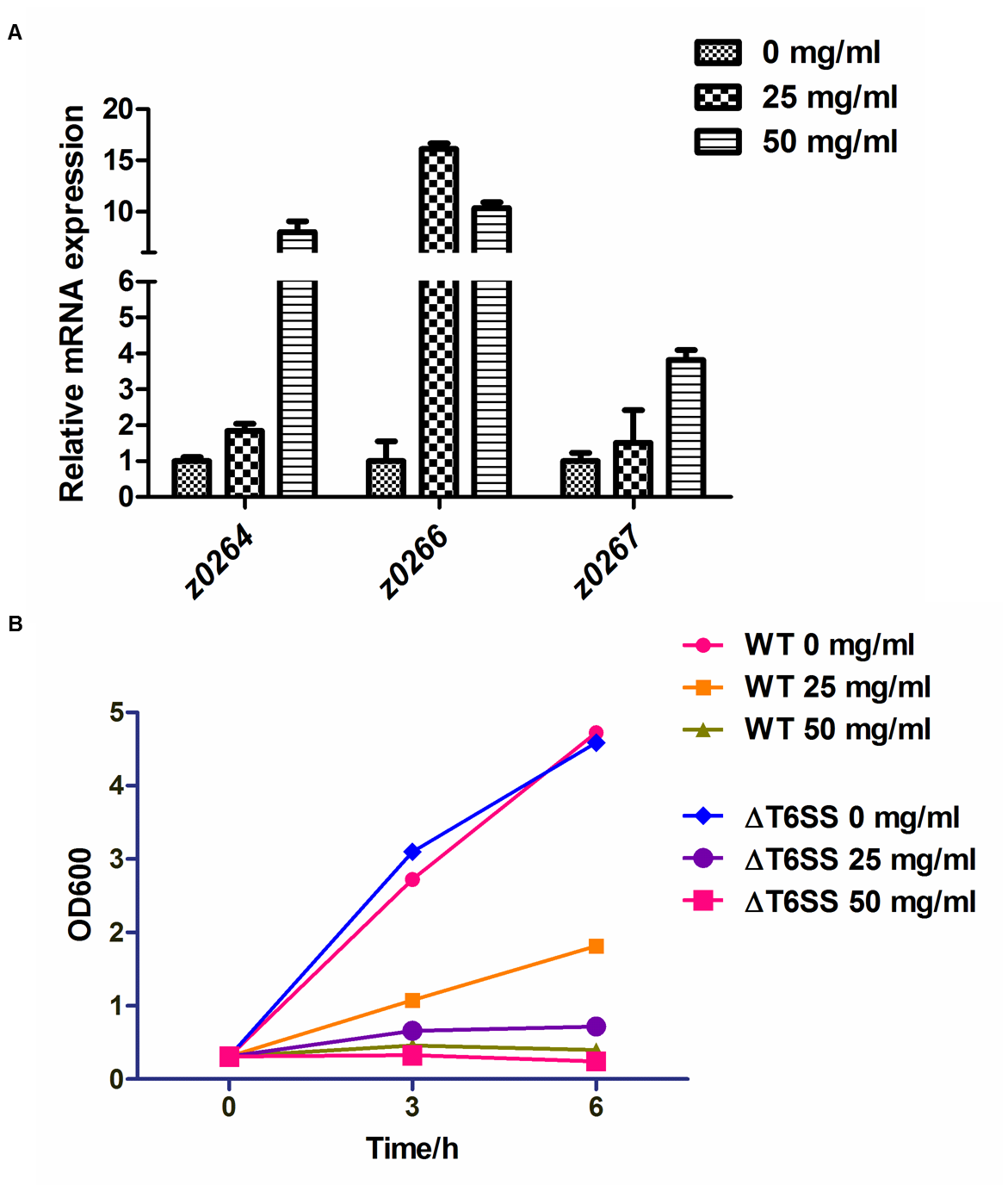

Supplement: S15 Fig — T6SS is involved in the survival of EHEC in bile salts. (A) Bile salts promote the transcriptional levels of T6SS genes of EHEC. Total RNA samples were isolated from EHEC cultured in LB broth supplemented with different concentrations of bovine bile salts at different time points at 37°C followed by qPCR analysis. The 16S rRNA was used as internal standard. Error bars represented SD from at least three independent experiments. (B) T6SS contributes to the growth of EHEC at the presence of bile salts. The overnight cultures were inoculated into 6 ml fresh LB broth supplemented with different concentrations of bovine bile salts at an initial OD600 of 0.3 at 37°C, and samples were collected at different time points for OD600 determination. (TIF) [file ppat.1006246.s019.tif]

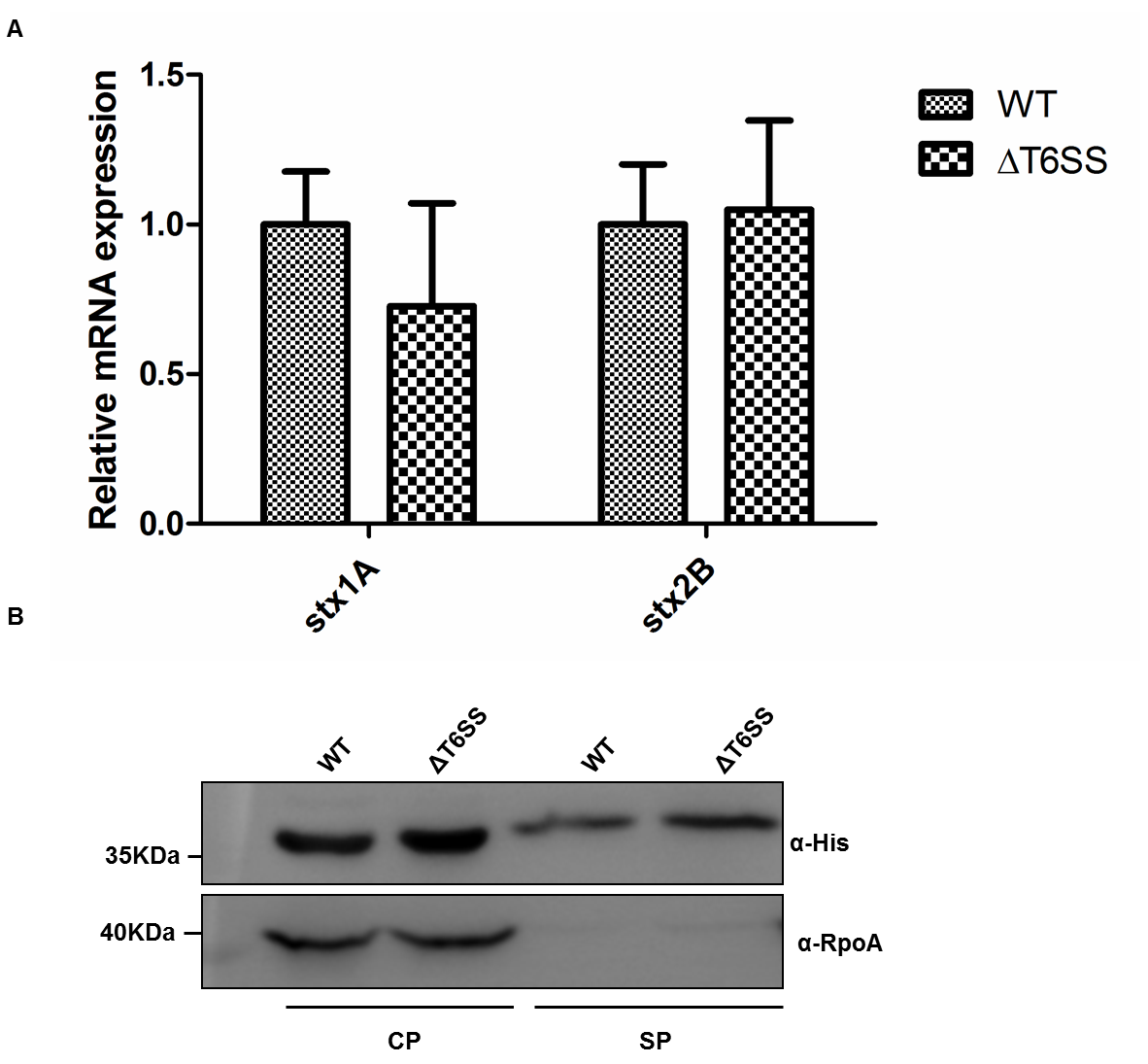

Supplement: S16 Fig — T6SS is not involved in the transcription and secretion of Shiga toxin. (A) T6SS is not involved in the transcription of Shiga toxins. Total RNA was isolated from WT and ΔT6SS at logarithmic phase (OD600 = 0.8) in LB broth at 37°C followed by the real-time qPCR analysis. The 16S rRNA was used as internal standard. (B) The absence of T6SS does not disturb the secretion of Shiga toxin. The WT and ΔT6SS bearing pQE80-stx2A with a His-tag sequence fusion at the C-termini were cultured to an OD600 = 1.0 in LB broth at 37°C. The pellet and supernatant fractions of the cultures were analyzed by Western blot using anti-His tag and anti-RpoA monoclonal antibodies. RpoA was used as an internal control. Three biological repeats were performed. (TIF) [file ppat.1006246.s020.tif]

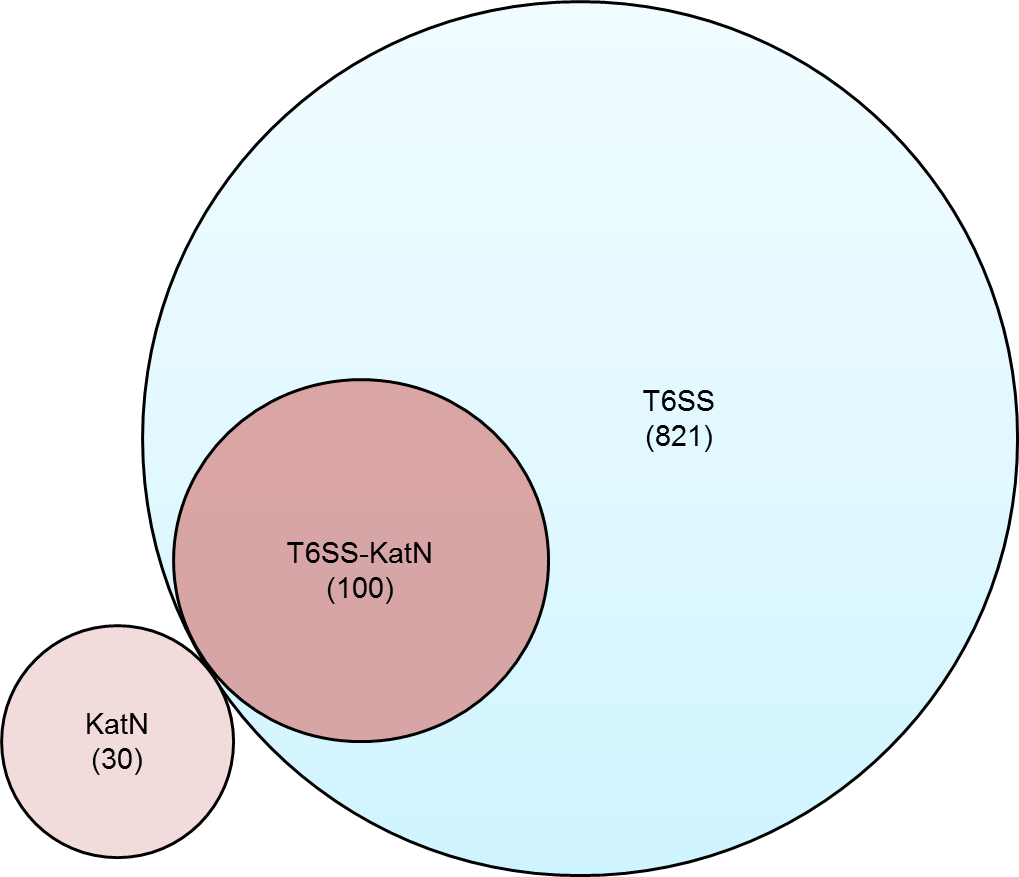

Supplement: S17 Fig — The distribution correlation between KatN and the T6SS. The amino acids sequence of KatN of EHEC was used to identify an additional 129 homologues by using BLASTP searches against the completely sequenced bacterial genomes with strict cutoffs (e-value less or equal to 0.0001 and identity higher or equal to 50%). Finally, 100 KatN homologues (76.9%) were determined to co-distribute with T6SS. In contrast, only 30 KatN homologues (23.1%) were inferred without co-presence of the T6SS gene cluster in the cognate bacterial genomes. Meanwhile, 821 bacterial genomes harboring T6SS gene clusters available in the archived SecReT6 database did not show the presence of KatN homologues. (TIF) [file ppat.1006246.s021.tif]
